# Supplementary figures and images for: A positive feedback loop between ZEB2 and ACSL4 regulates lipid metabolism to promote breast cancer metastasis
Source: eLife. 2023 Dec 11;12:RP87510. doi: 10.7554/eLife.87510 (PMC10712958; doi:10.7554/eLife.87510)

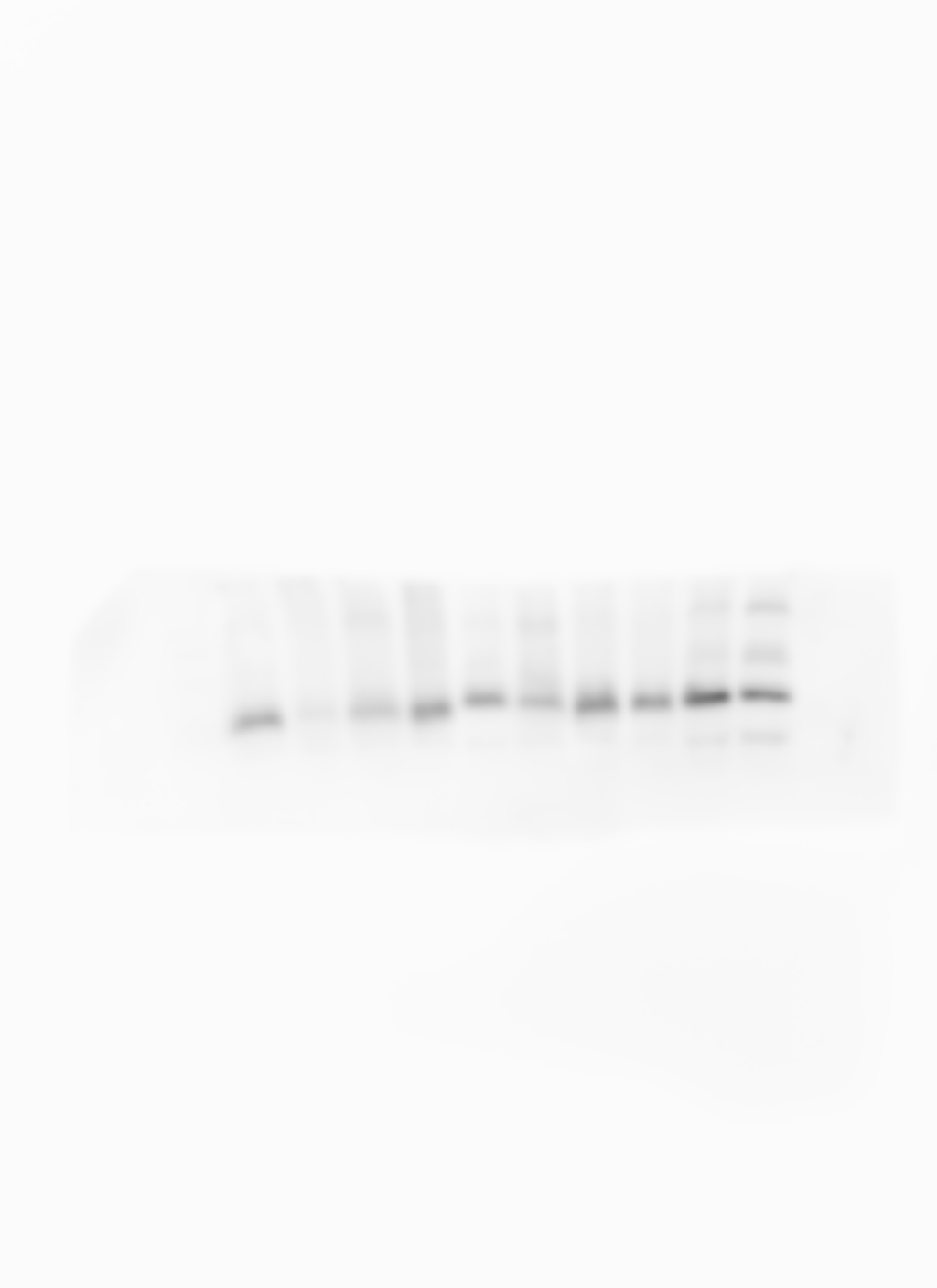

Supplement: Figure 1—source data 3. [file elife-87510-fig1-data3.zip › figure1-source-data/acsl4-patiant .tif]

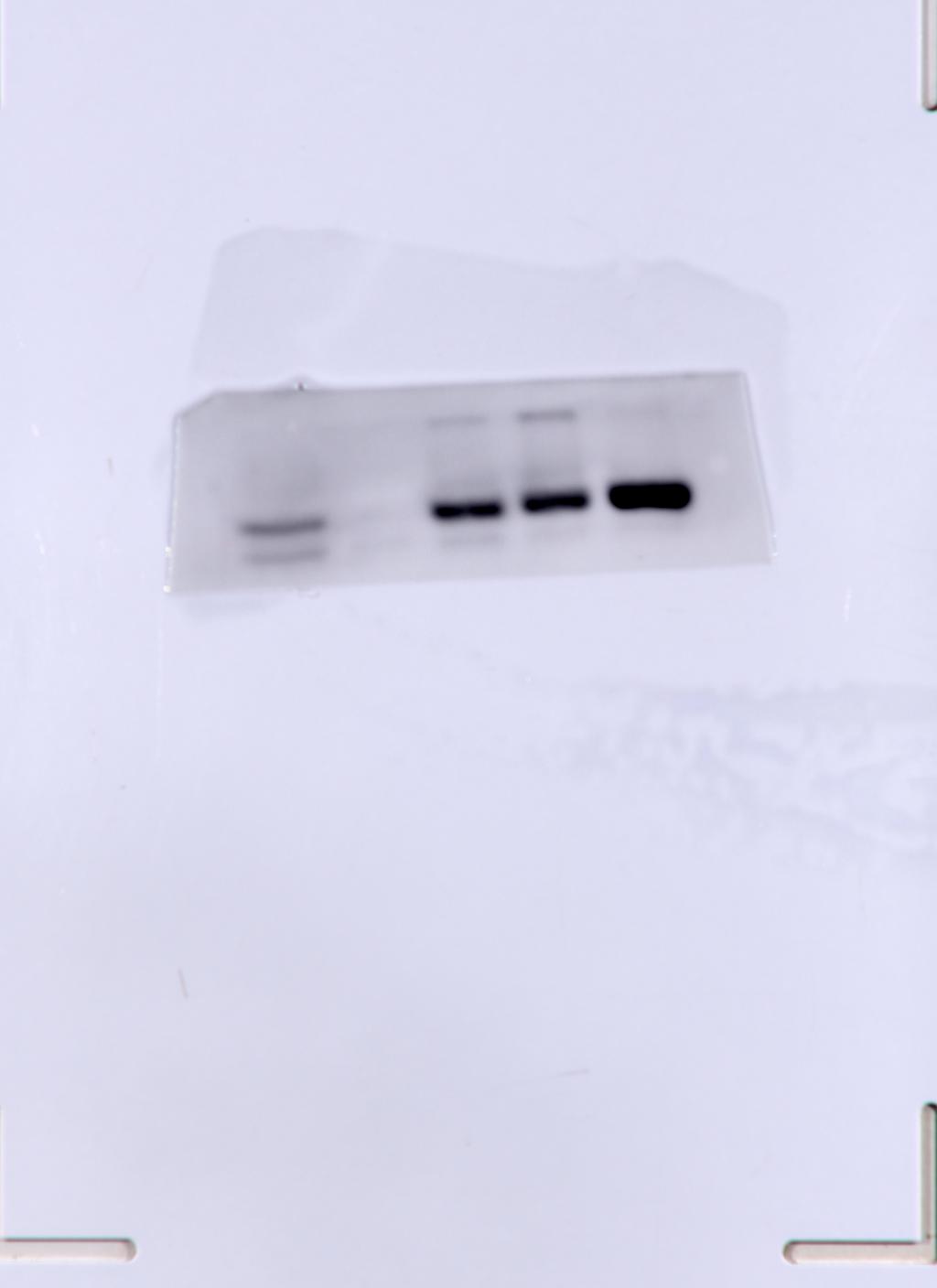

Supplement: Figure 1—source data 3. [file elife-87510-fig1-data3.zip › figure1-source-data/figure1G-ACSL4.jpg]

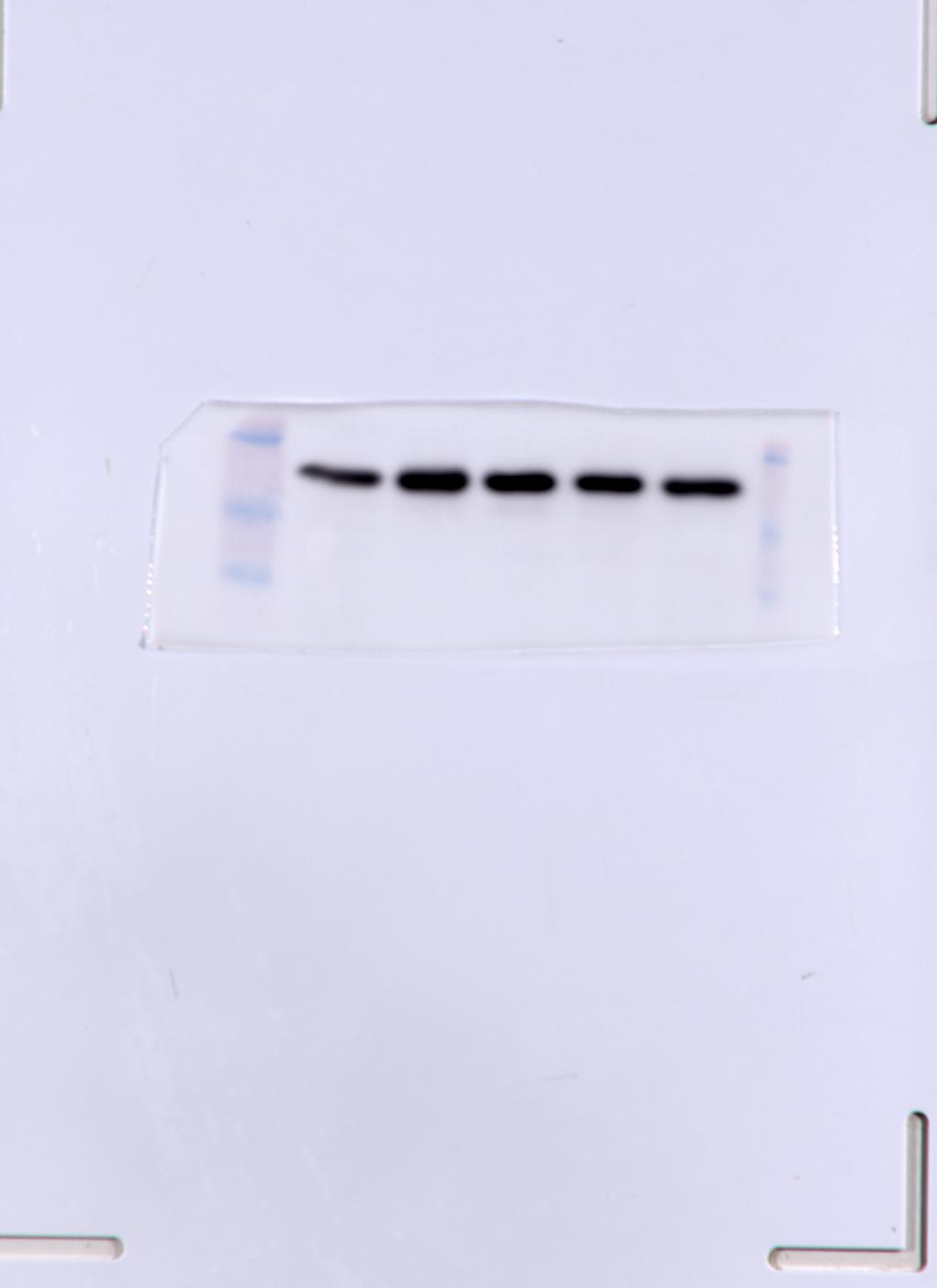

Supplement: Figure 1—source data 3. [file elife-87510-fig1-data3.zip › figure1-source-data/figure1G-gapdh1.jpg]

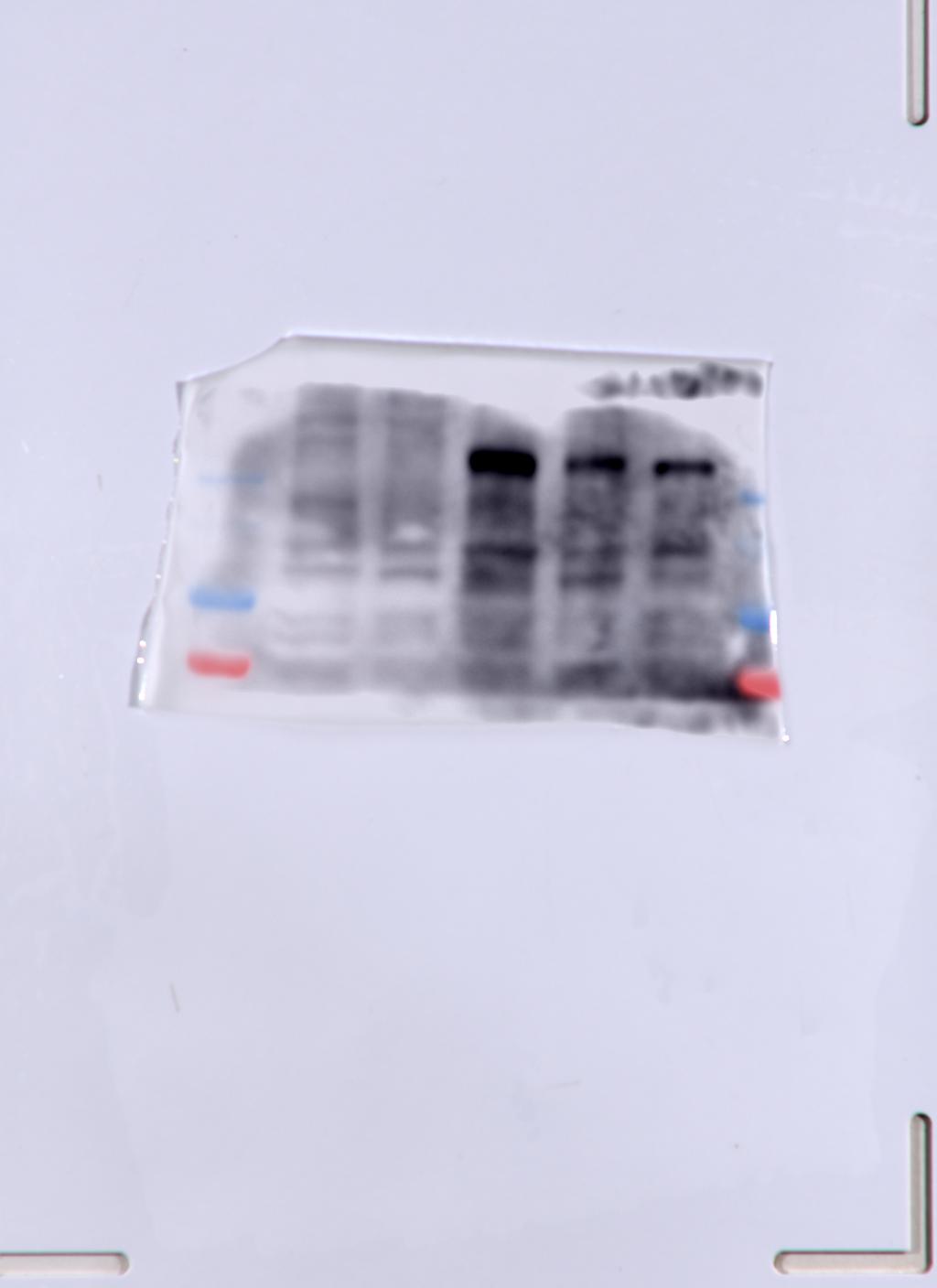

Supplement: Figure 1—source data 3. [file elife-87510-fig1-data3.zip › figure1-source-data/figure1G-ZEB2.jpg]

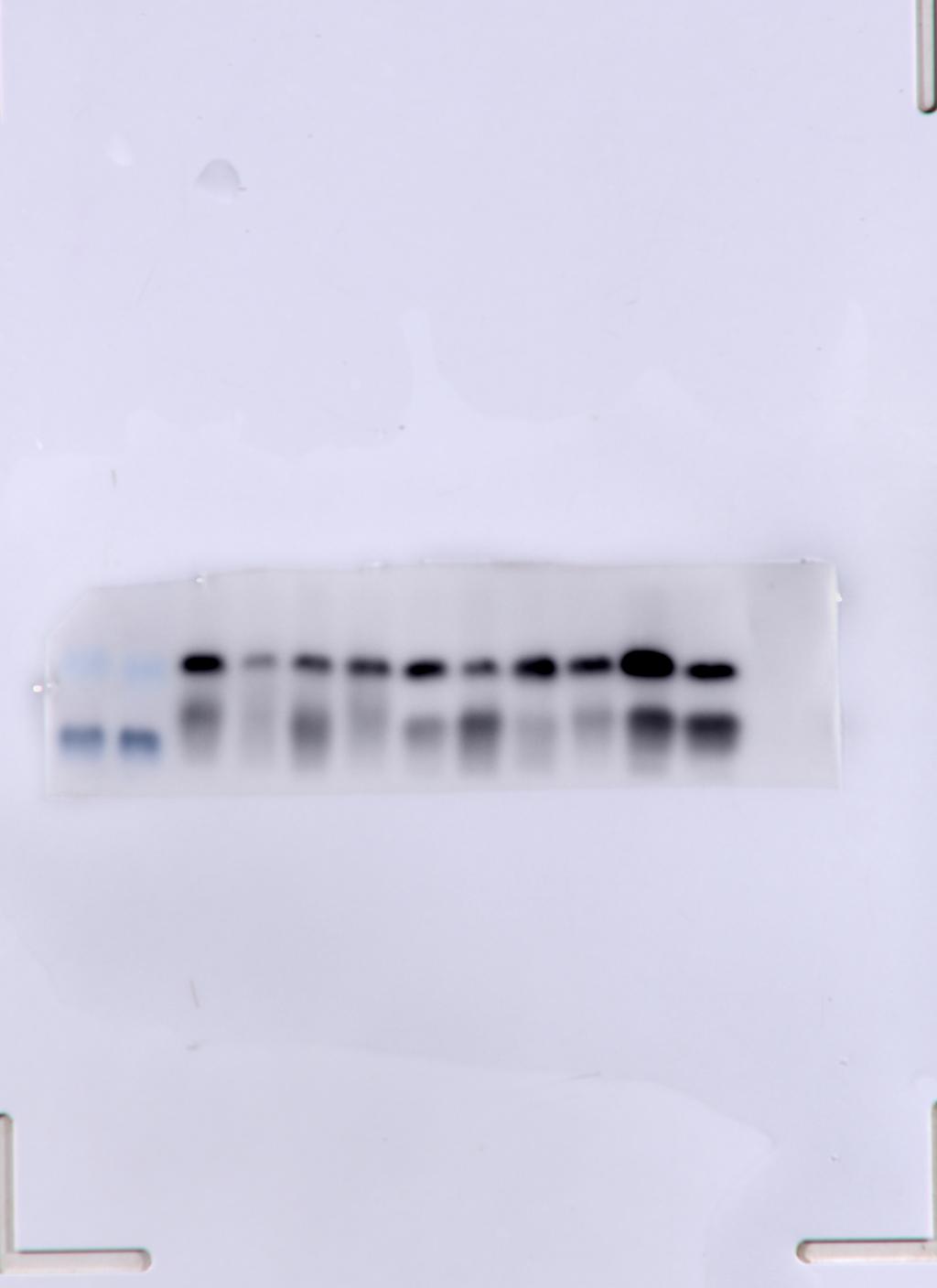

Supplement: Figure 1—source data 3. [file elife-87510-fig1-data3.zip › figure1-source-data/gapdh-patient.jpg]

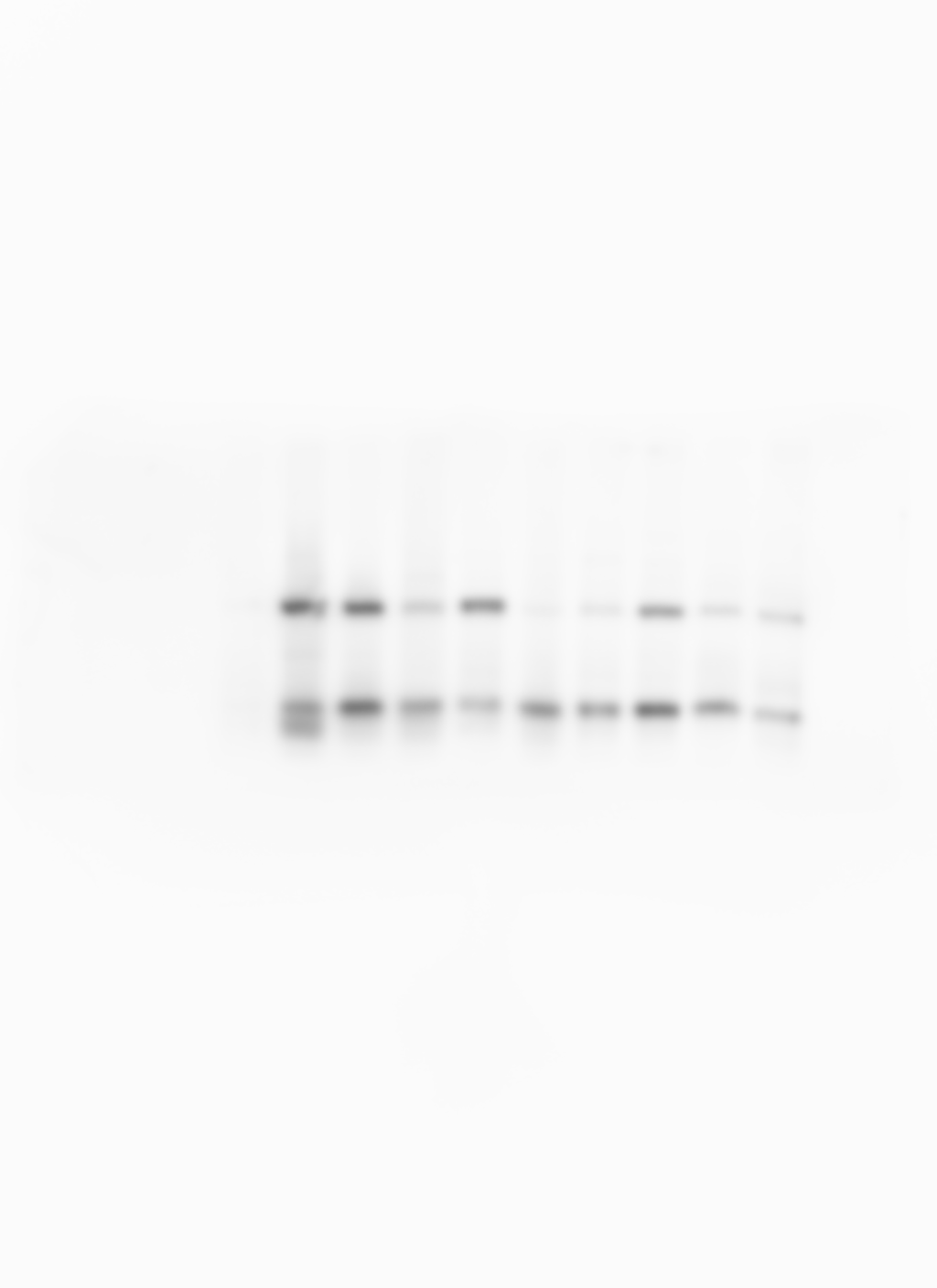

Supplement: Figure 1—source data 3. [file elife-87510-fig1-data3.zip › figure1-source-data/Zeb-2-patient .tif]

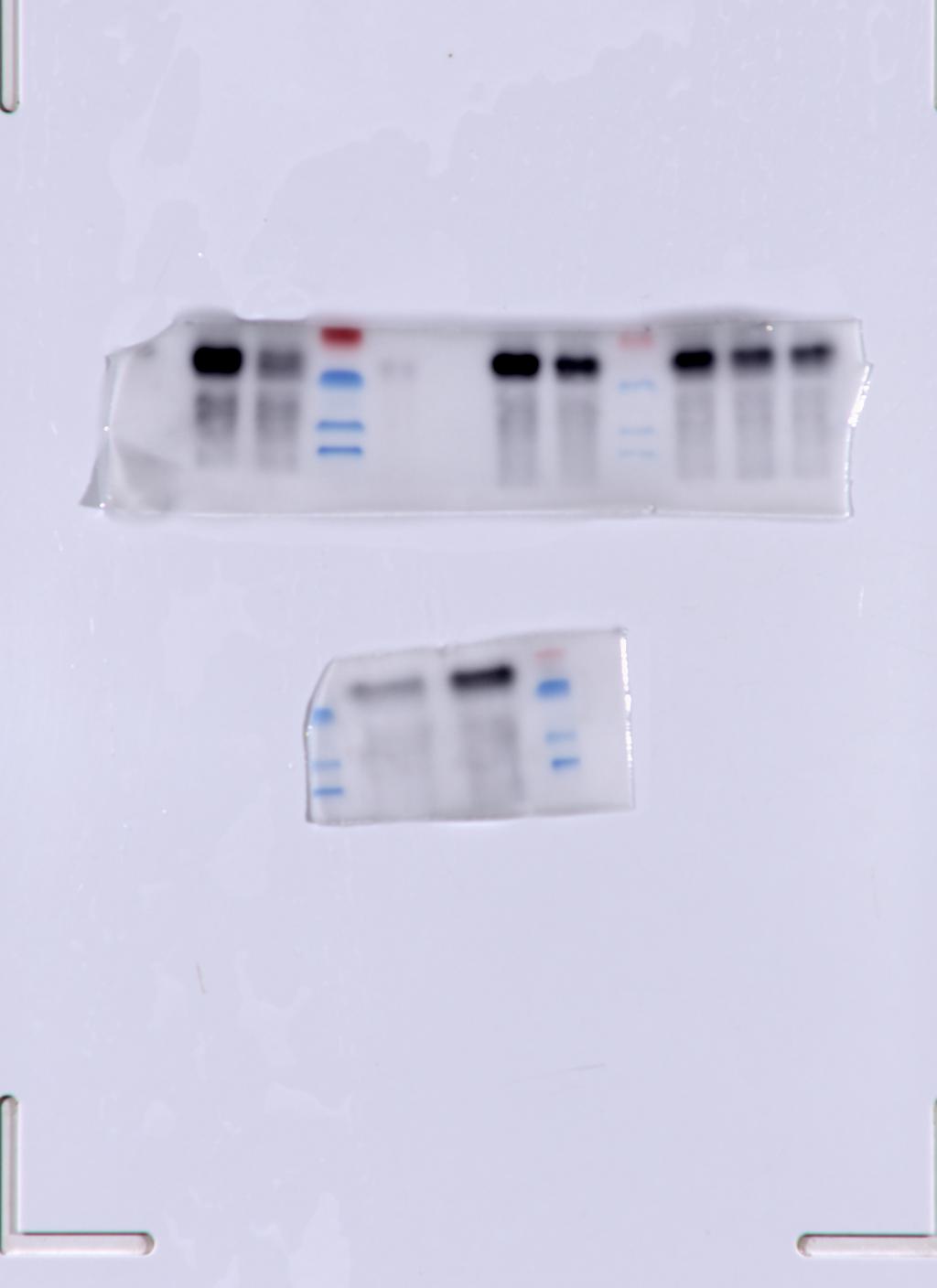

Supplement: Figure 2—source data 2. [file elife-87510-fig2-data2.zip › figure2-source-data/figure2A-2H-acsl4 .jpg]

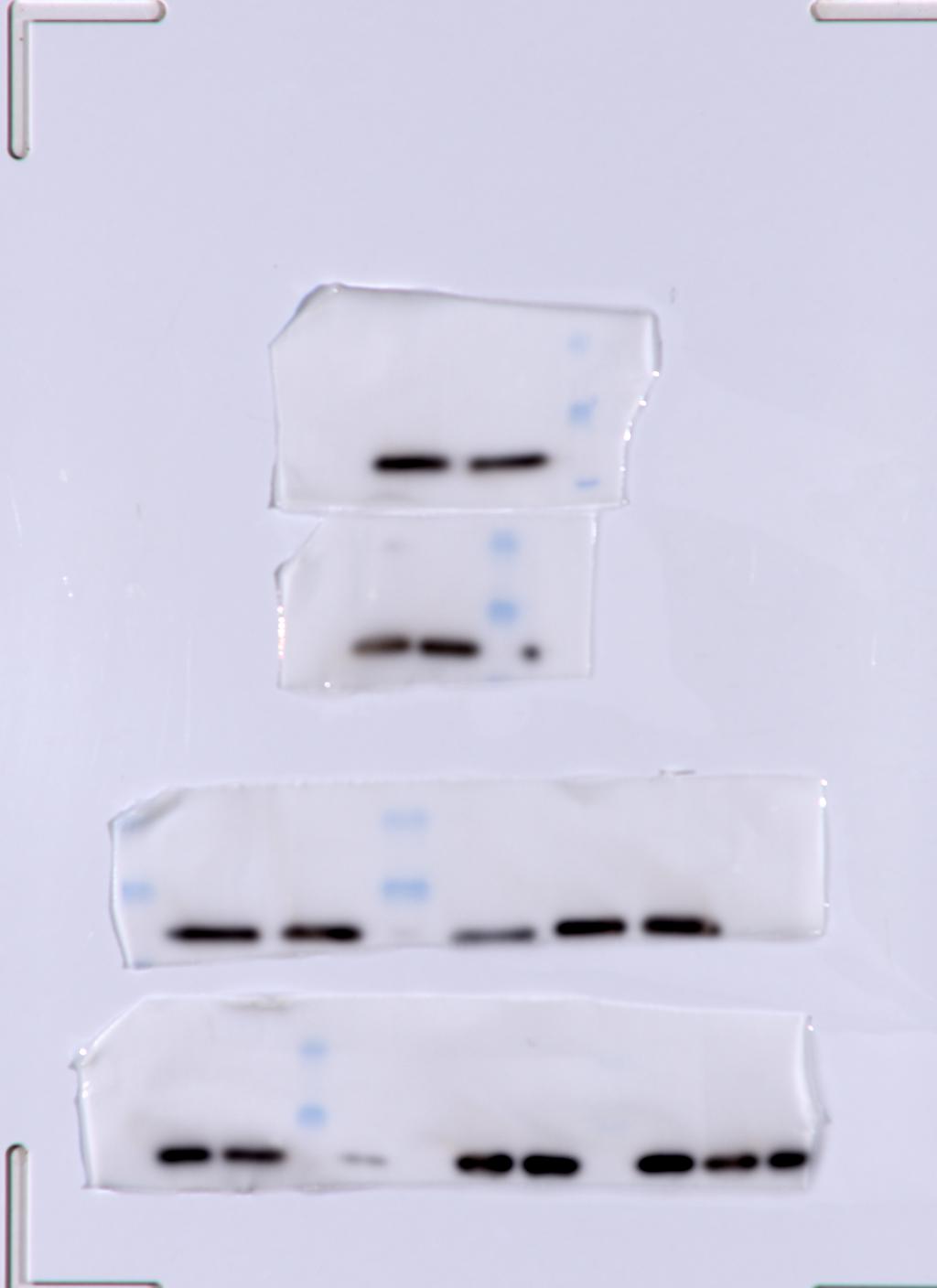

Supplement: Figure 2—source data 2. [file elife-87510-fig2-data2.zip › figure2-source-data/figure2A-gapdh.jpg]

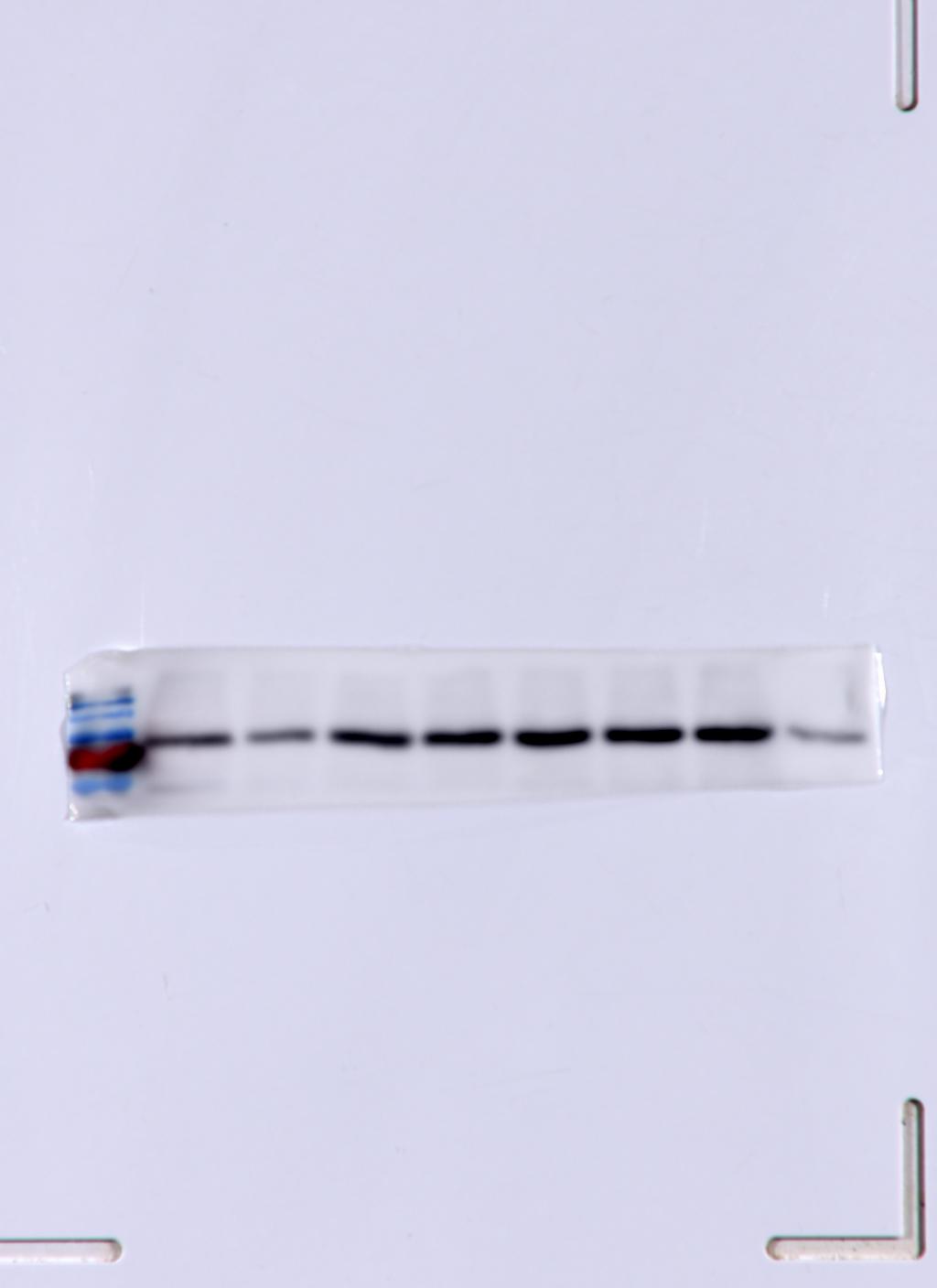

Supplement: Figure 2—source data 2. [file elife-87510-fig2-data2.zip › figure2-source-data/figure2C-acsl4 .jpg]

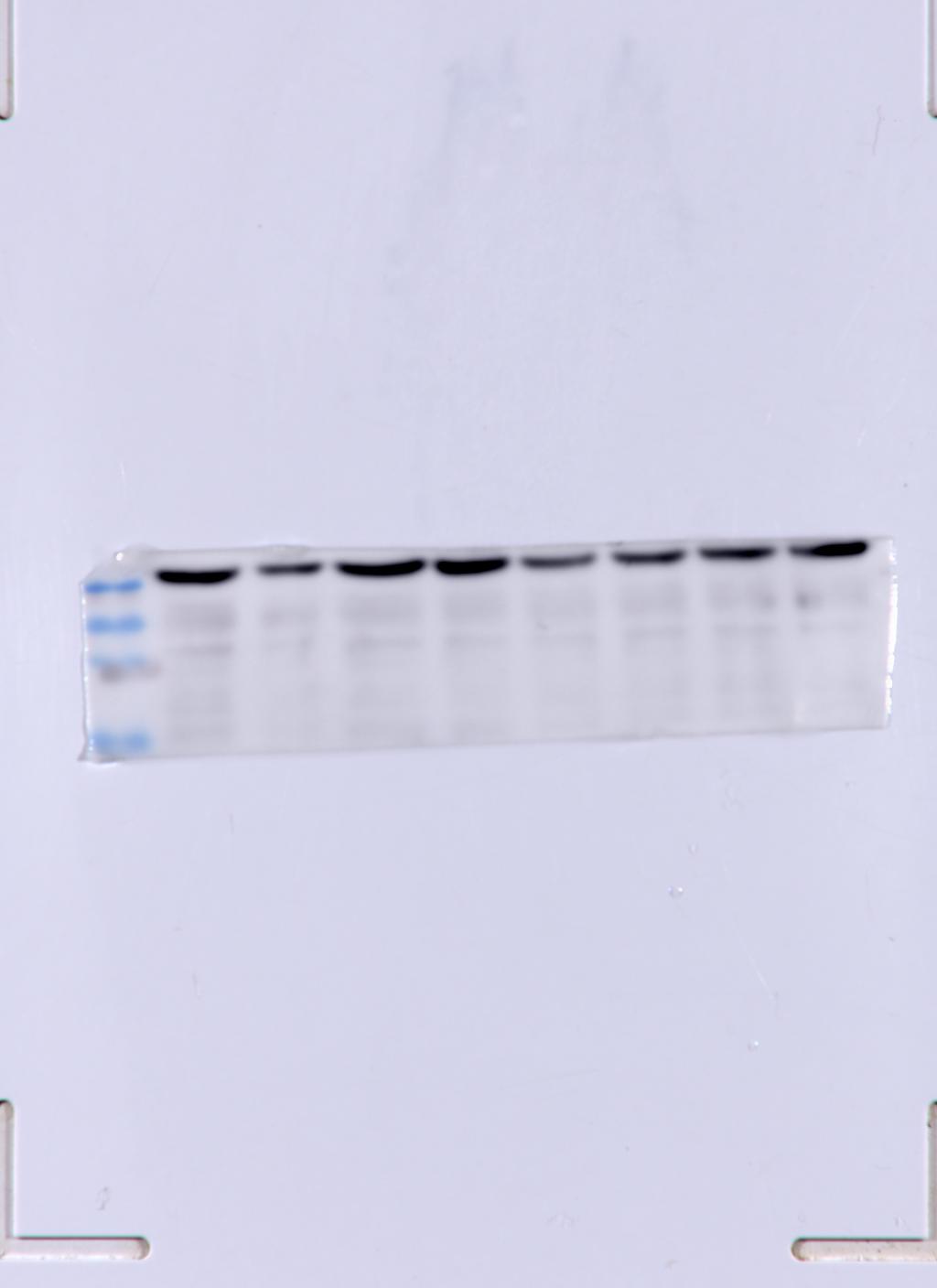

Supplement: Figure 2—source data 2. [file elife-87510-fig2-data2.zip › figure2-source-data/figure2C-actin.jpg]

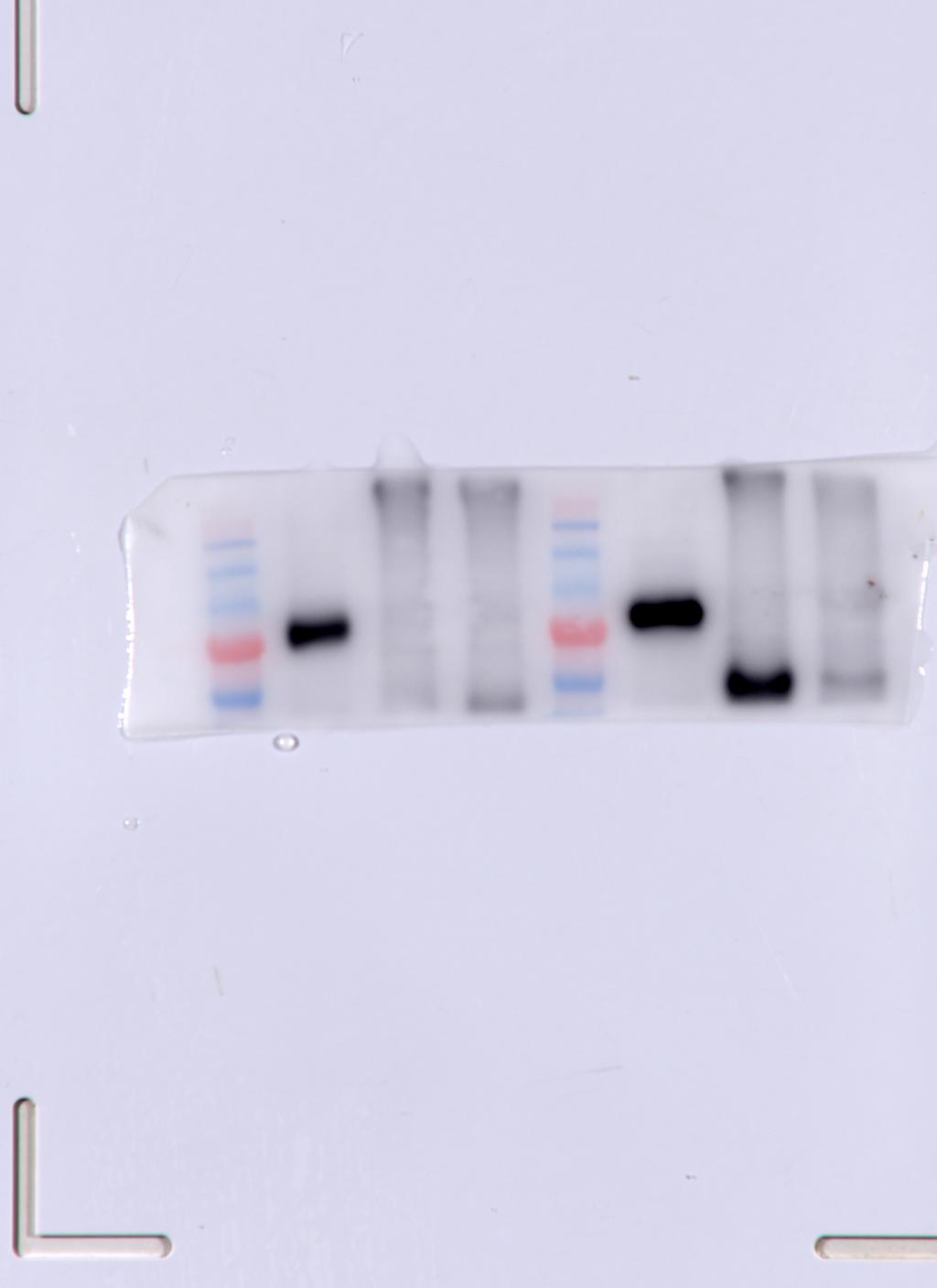

Supplement: Figure 2—source data 2. [file elife-87510-fig2-data2.zip › figure2-source-data/Figure2F-ACSL4.jpg]

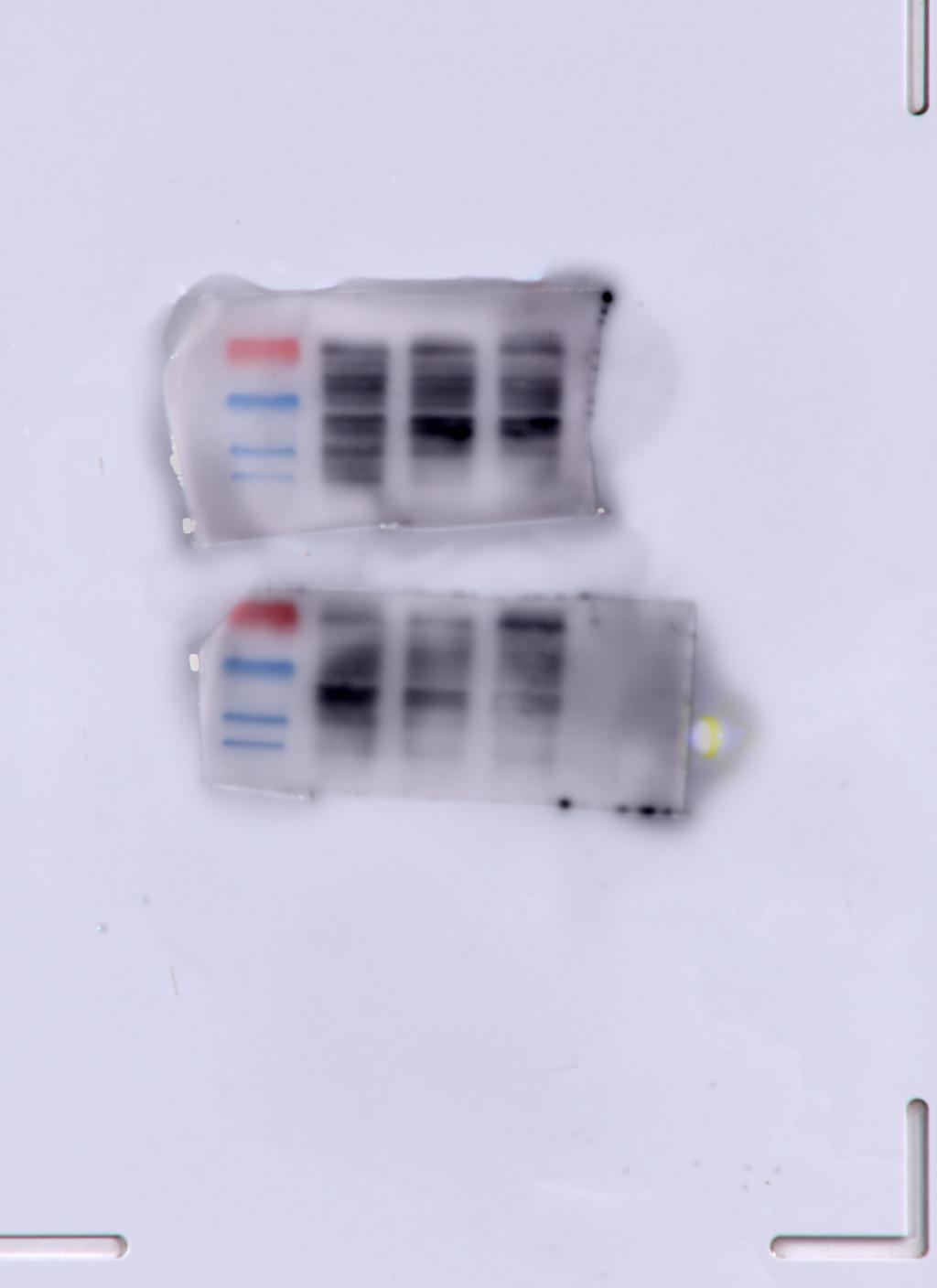

Supplement: Figure 2—source data 2. [file elife-87510-fig2-data2.zip › figure2-source-data/figure2F-E-cadherin(upper) N-cadherin(lower).jpg]

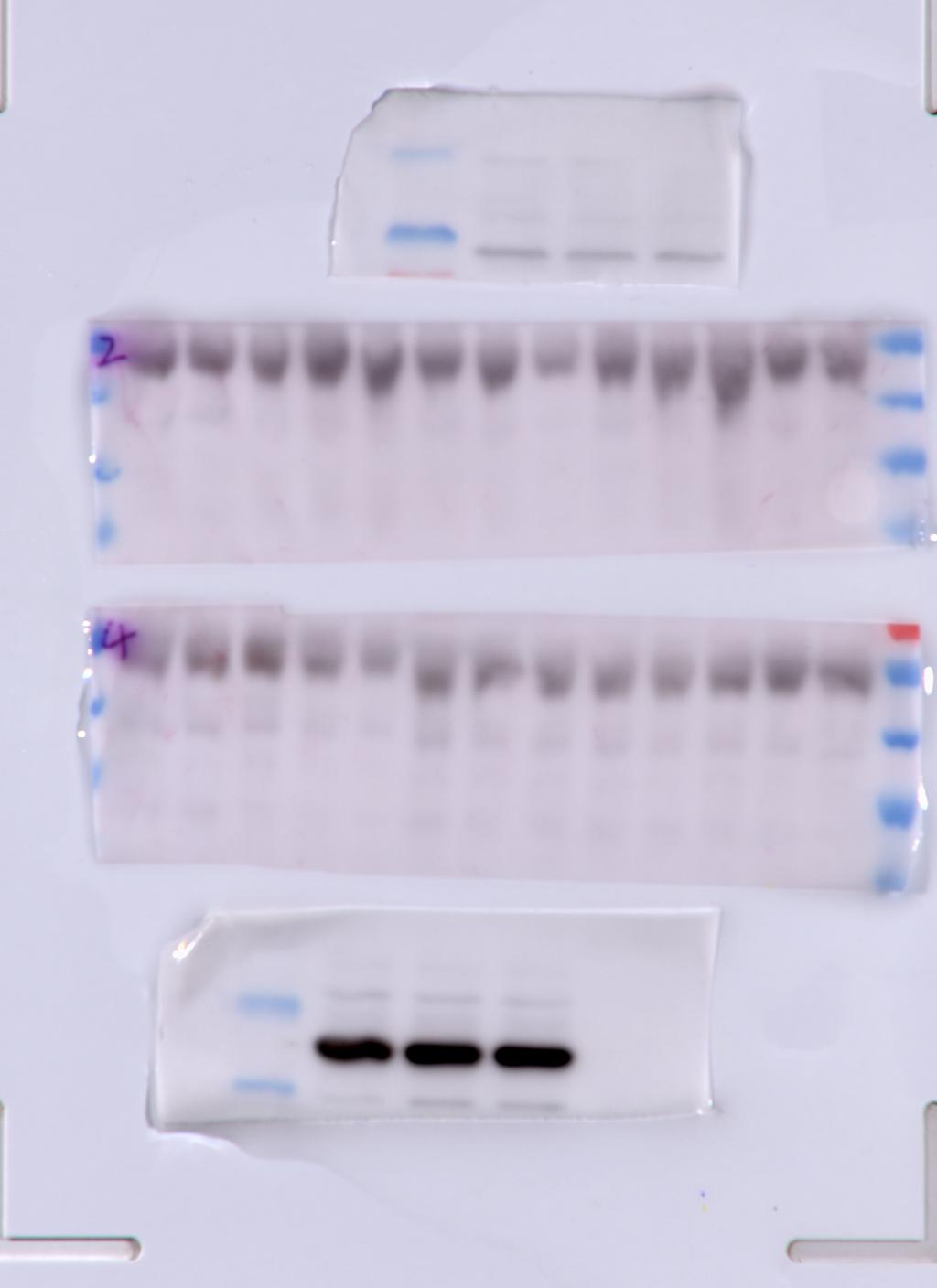

Supplement: Figure 2—source data 2. [file elife-87510-fig2-data2.zip › figure2-source-data/figure2F-gapdh.jpg]

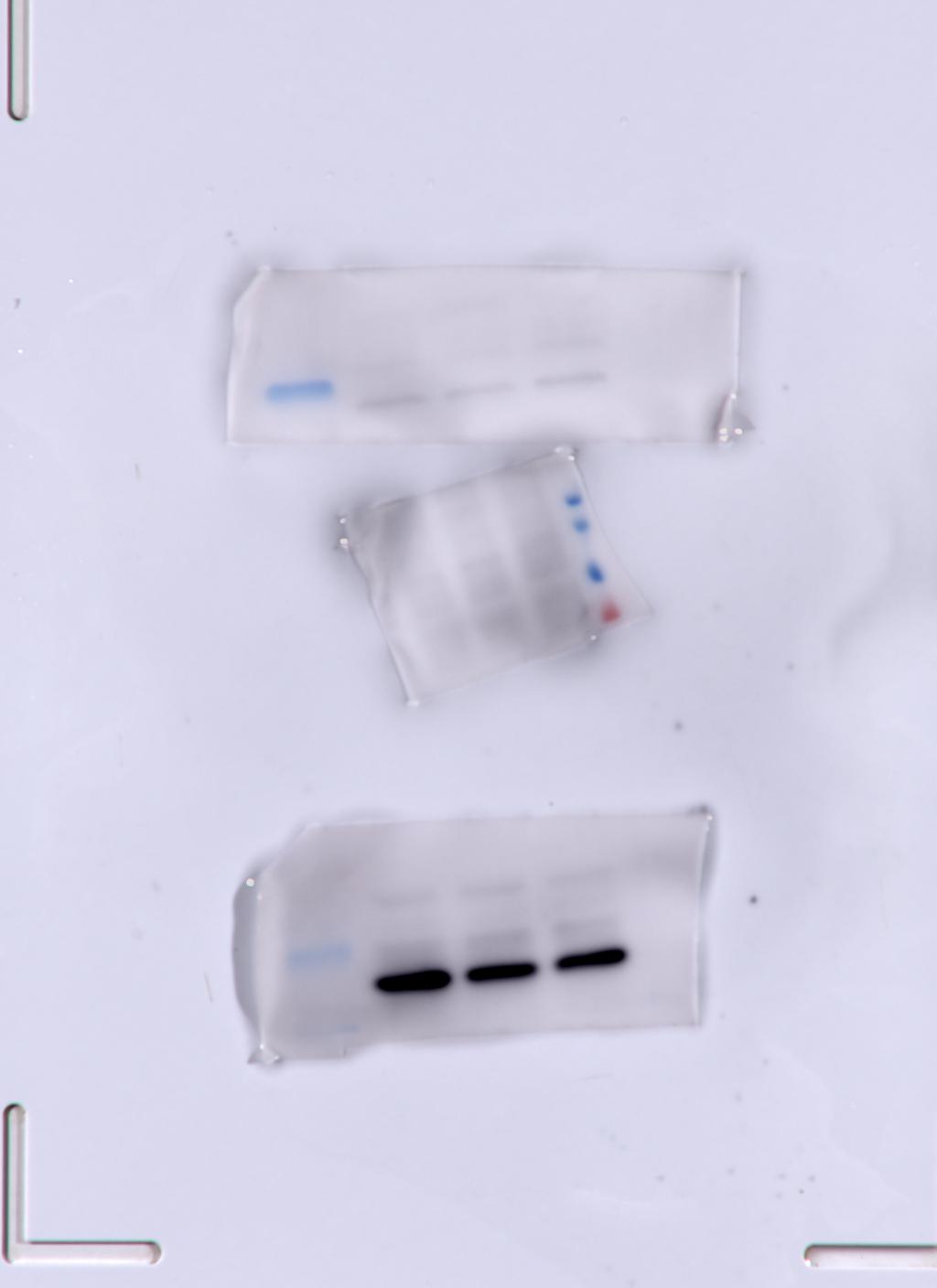

Supplement: Figure 2—source data 2. [file elife-87510-fig2-data2.zip › figure2-source-data/figure2F-Vimentin .jpg]

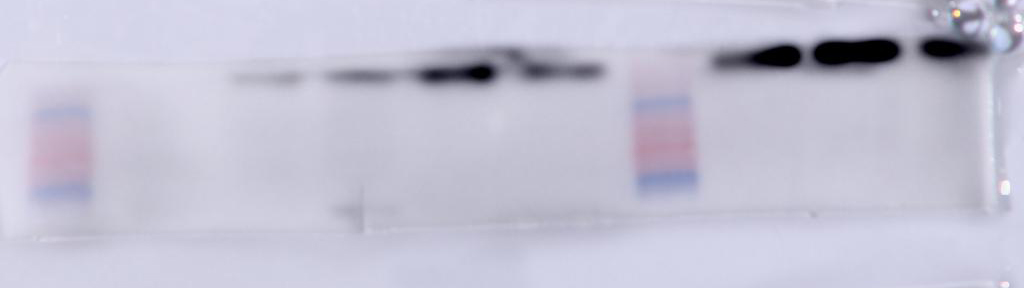

Supplement: Figure 2—source data 2. [file elife-87510-fig2-data2.zip › figure2-source-data/Figure2F-ZEB2.jpg]

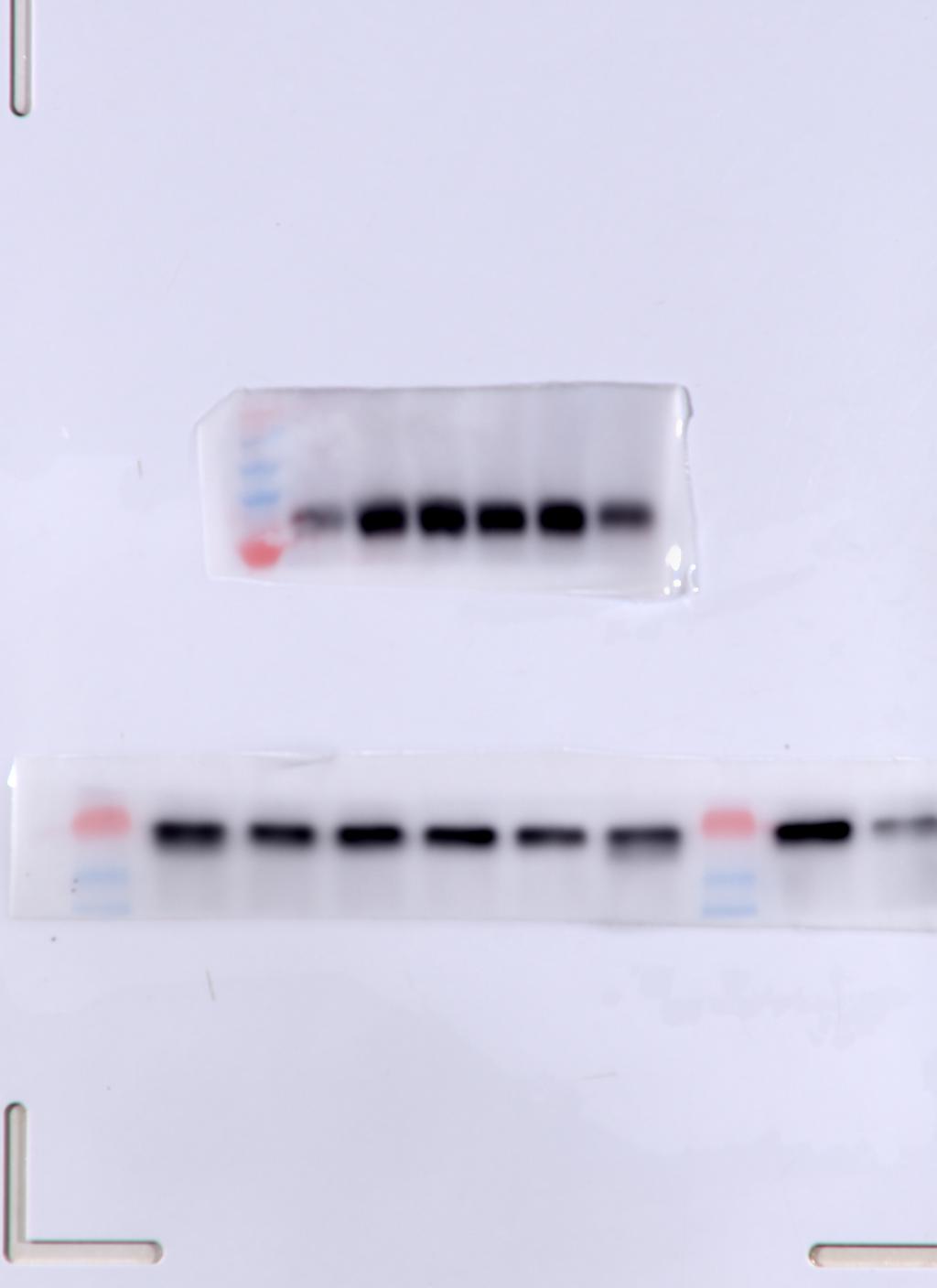

Supplement: Figure 2—source data 2. [file elife-87510-fig2-data2.zip › figure2-source-data/Figure2H-acsl4 .jpg]

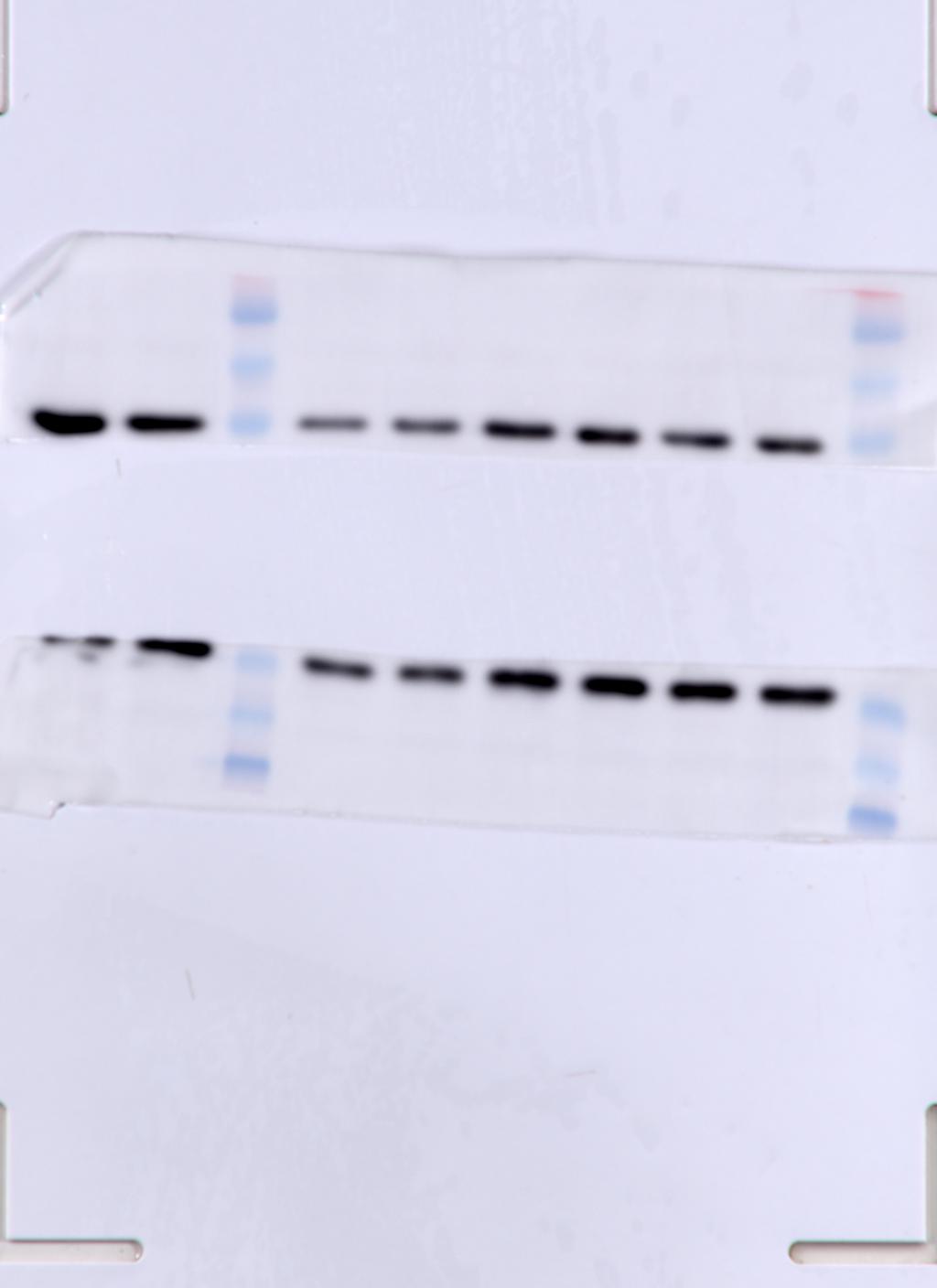

Supplement: Figure 2—source data 2. [file elife-87510-fig2-data2.zip › figure2-source-data/Figure2H-gapdh(lower part) .jpg]

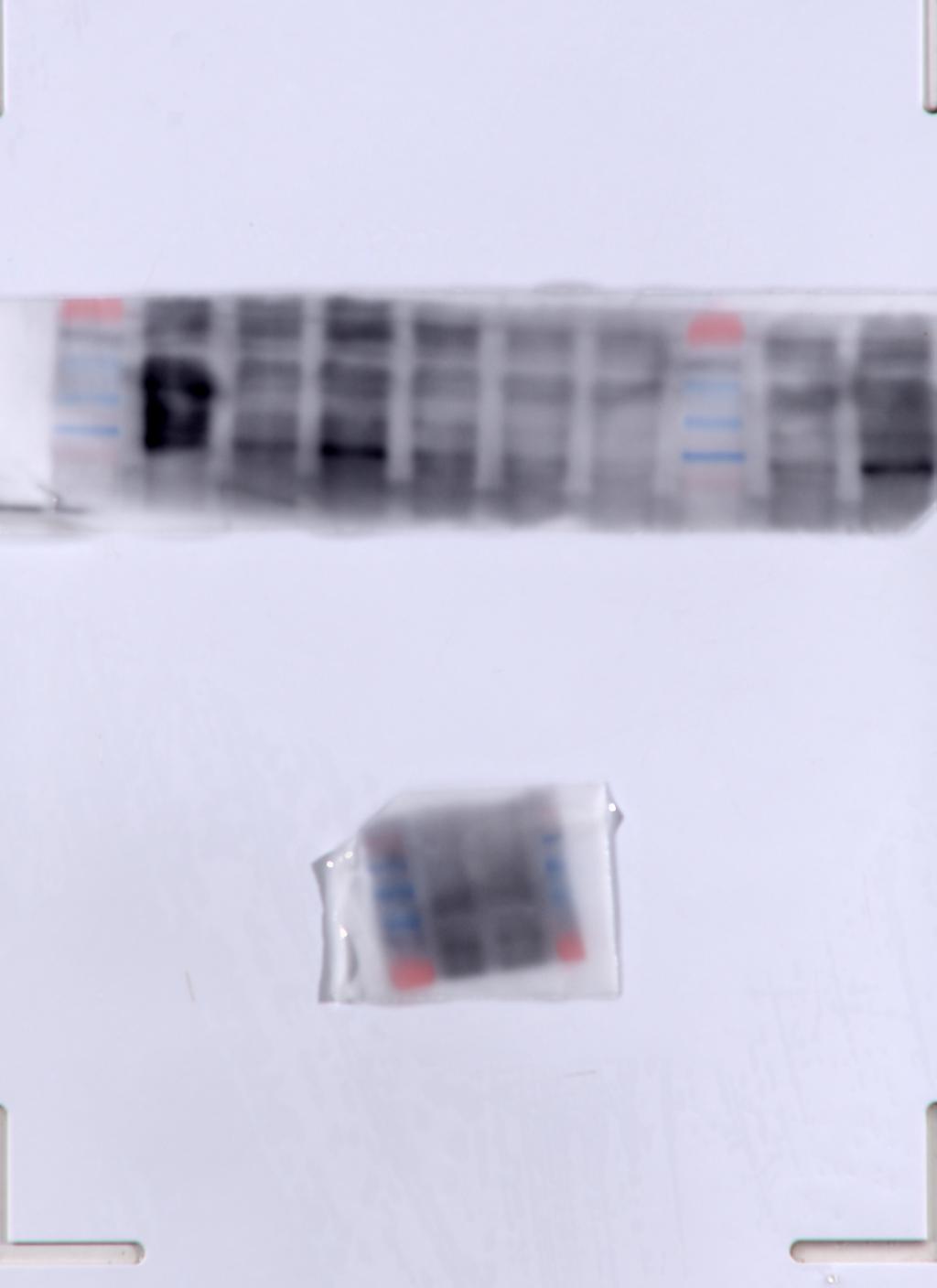

Supplement: Figure 2—source data 2. [file elife-87510-fig2-data2.zip › figure2-source-data/Figure2H-ZeEB2.jpg]

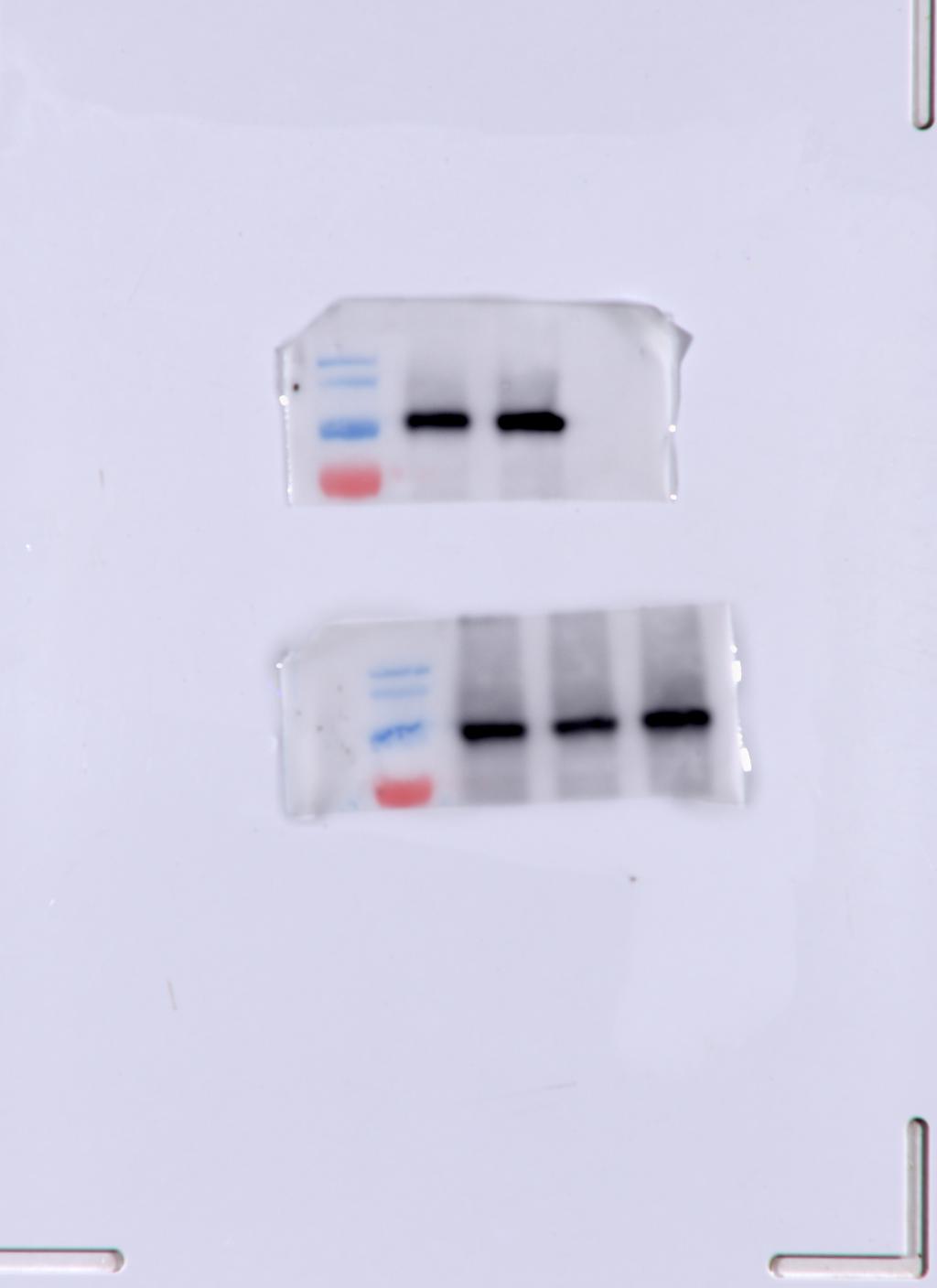

Supplement: Figure 5—source data 1. [file elife-87510-fig5-data1.zip › figure5-source-data/CPT1A .jpg]

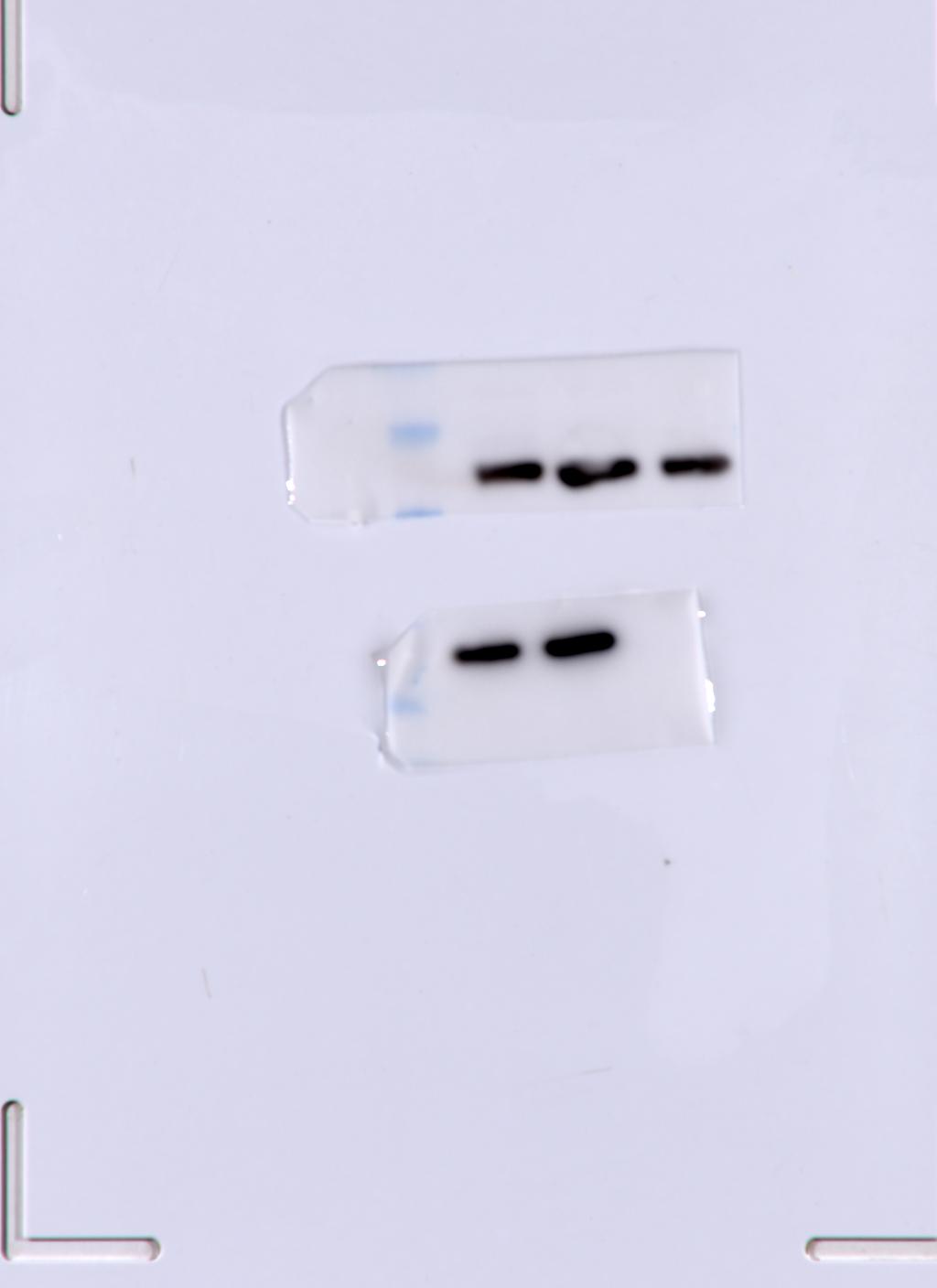

Supplement: Figure 5—source data 1. [file elife-87510-fig5-data1.zip › figure5-source-data/gapdh.jpg]

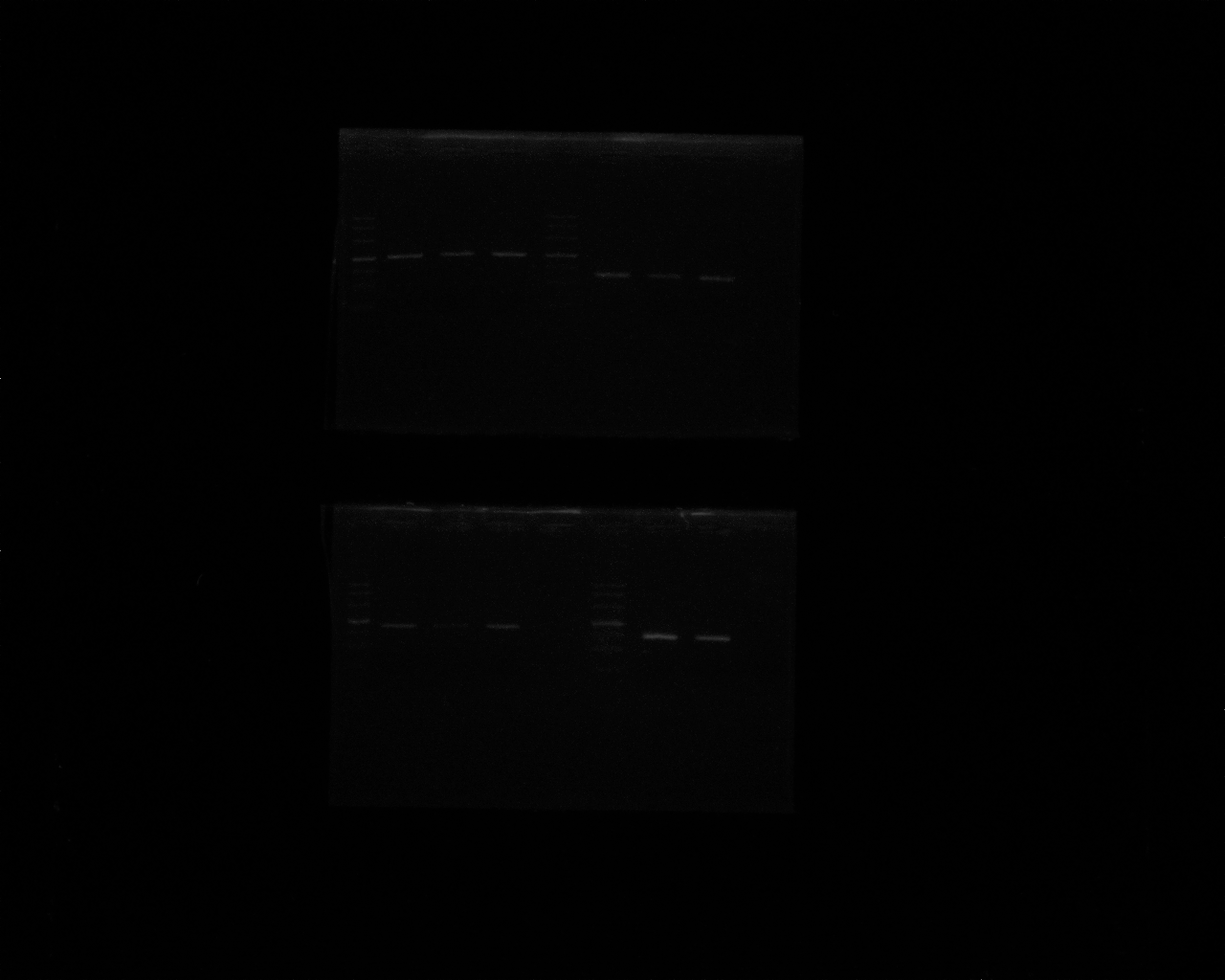

Supplement: Figure 6—source data 1. [file elife-87510-fig6-data1.zip › figure6-source-data/chip2021-8-6-1.png]

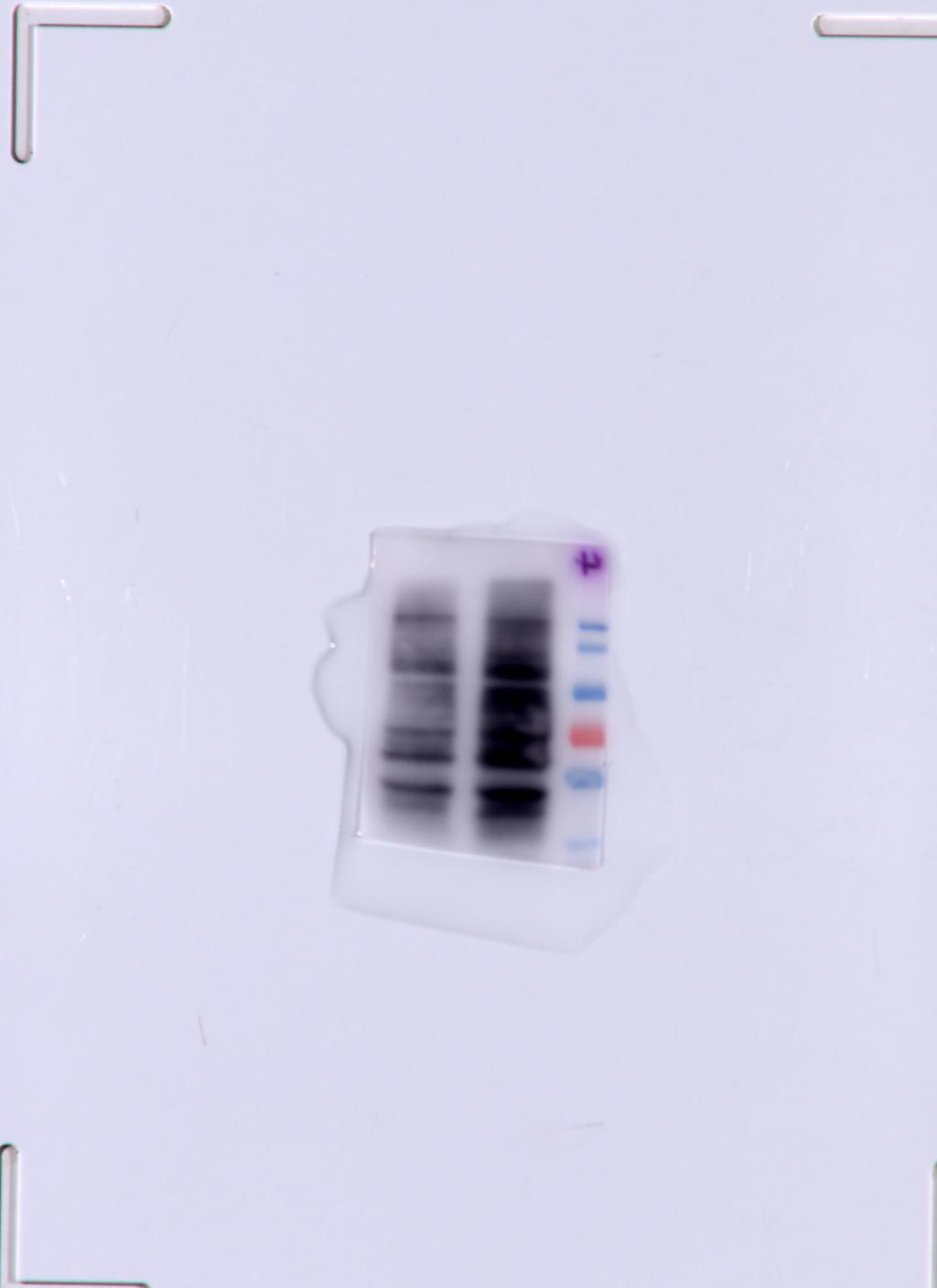

Supplement: Figure 6—source data 1. [file elife-87510-fig6-data1.zip › figure6-source-data/figure6-zeb2.jpg]

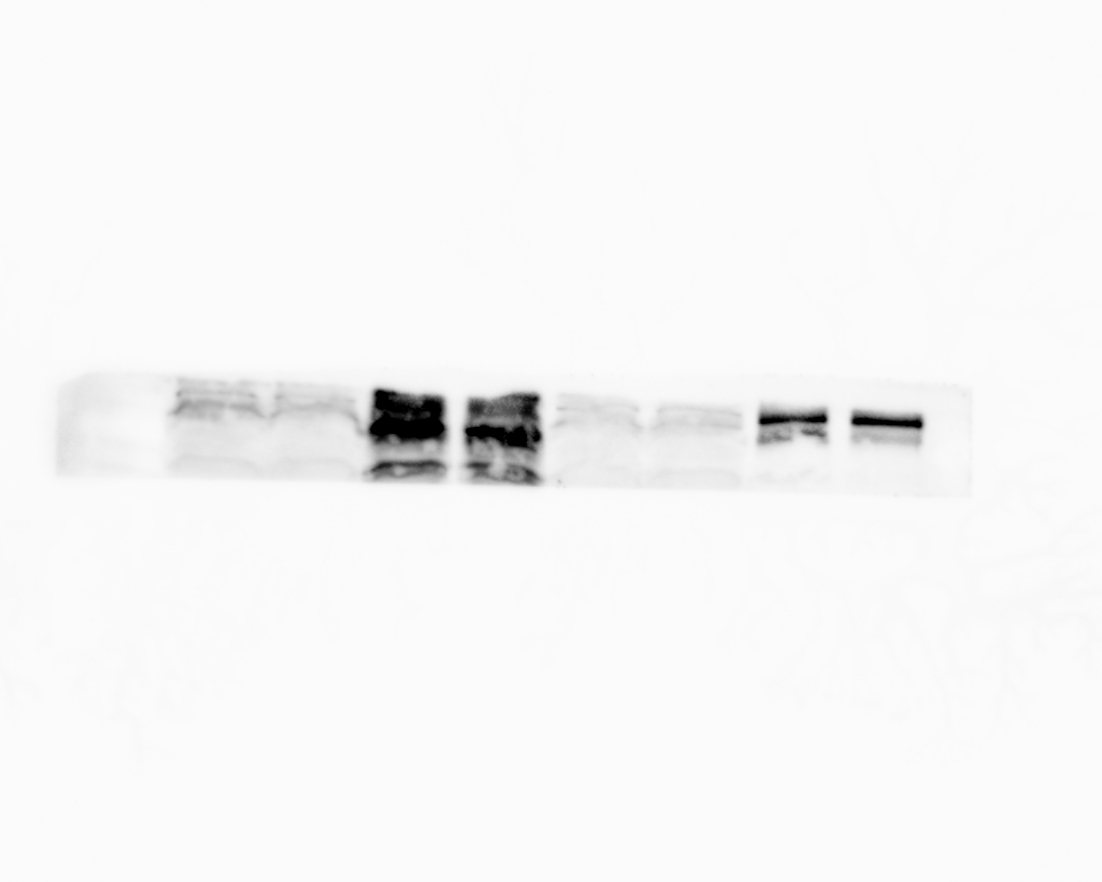

Supplement: Figure 7—source data 1. [file elife-87510-fig7-data1.zip › figure7-soure-data/ACSL4input.jpg]

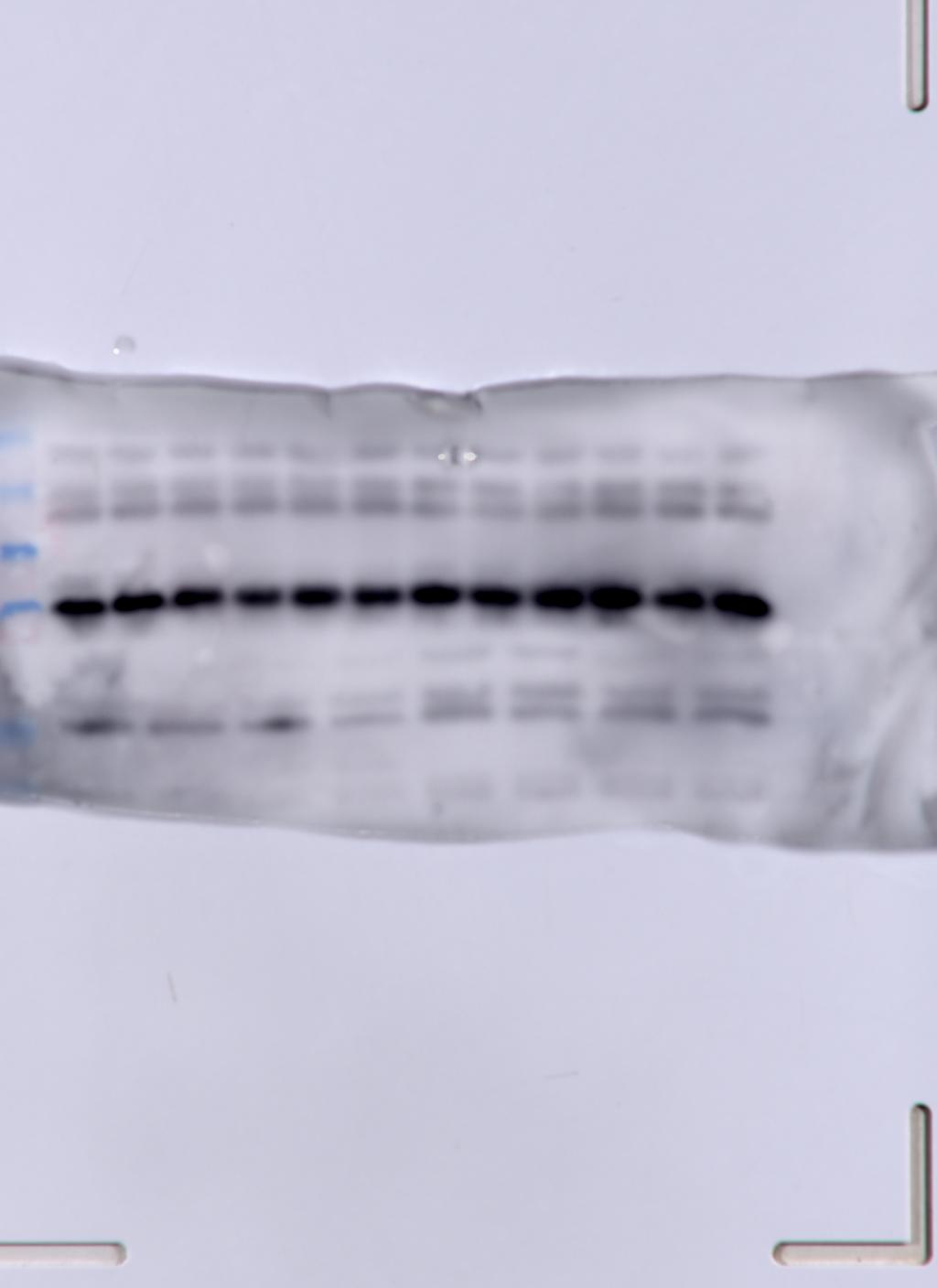

Supplement: Figure 7—source data 1. [file elife-87510-fig7-data1.zip › figure7-soure-data/Figuer7G-Gapdh.jpg]

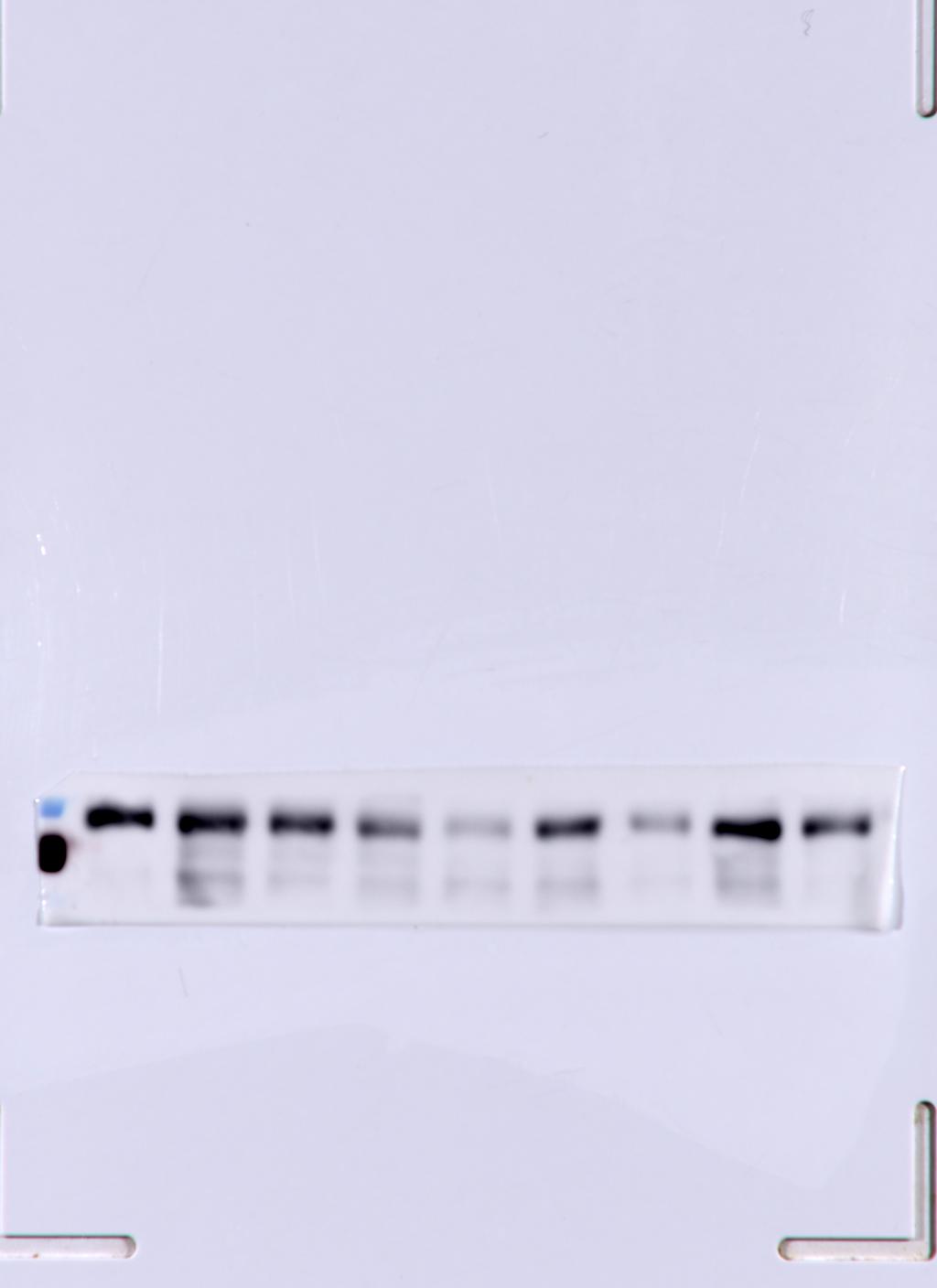

Supplement: Figure 7—source data 1. [file elife-87510-fig7-data1.zip › figure7-soure-data/Figure7B-ACSL4 .jpg]

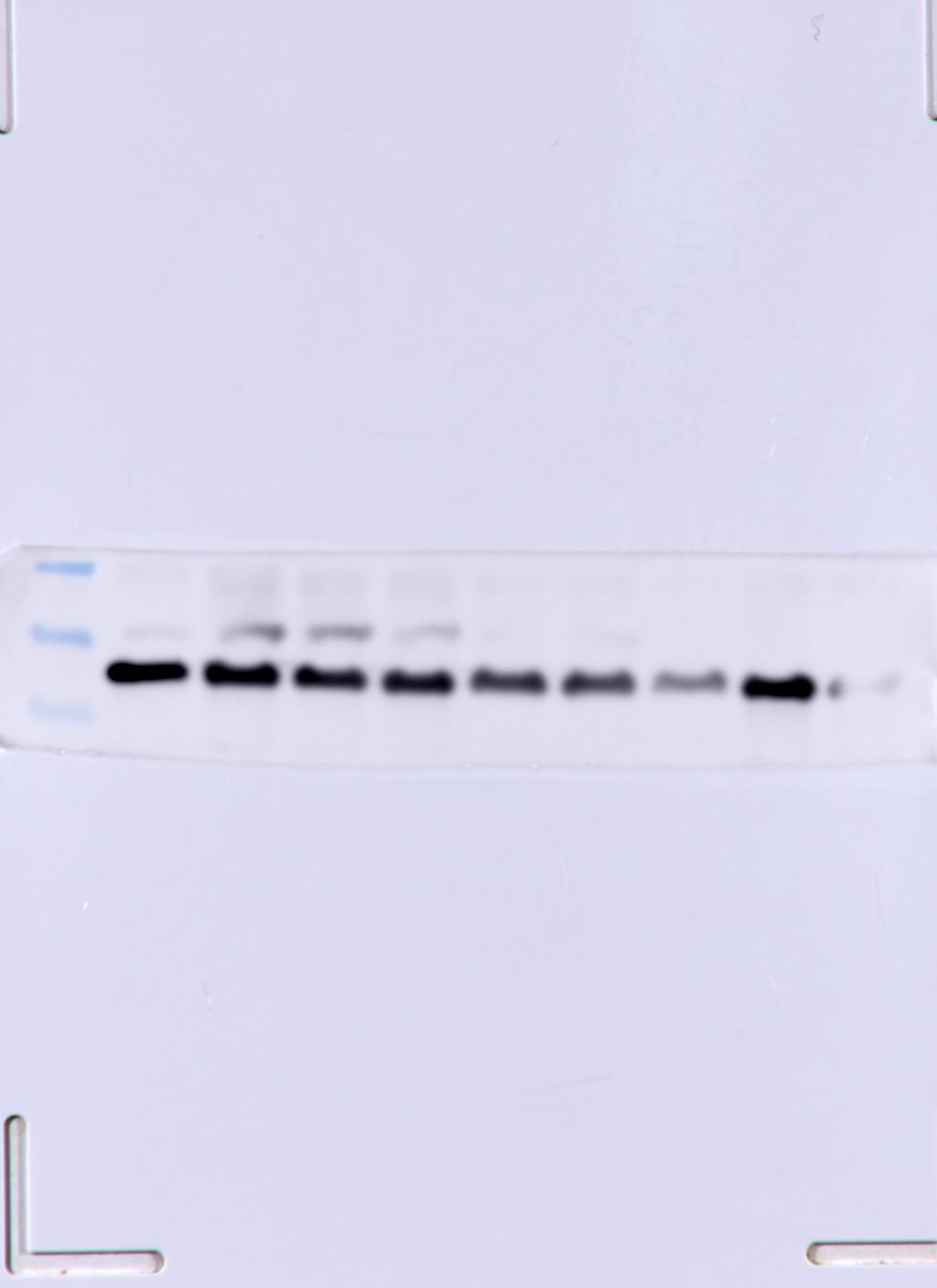

Supplement: Figure 7—source data 1. [file elife-87510-fig7-data1.zip › figure7-soure-data/Figure7B-gapdh .jpg]

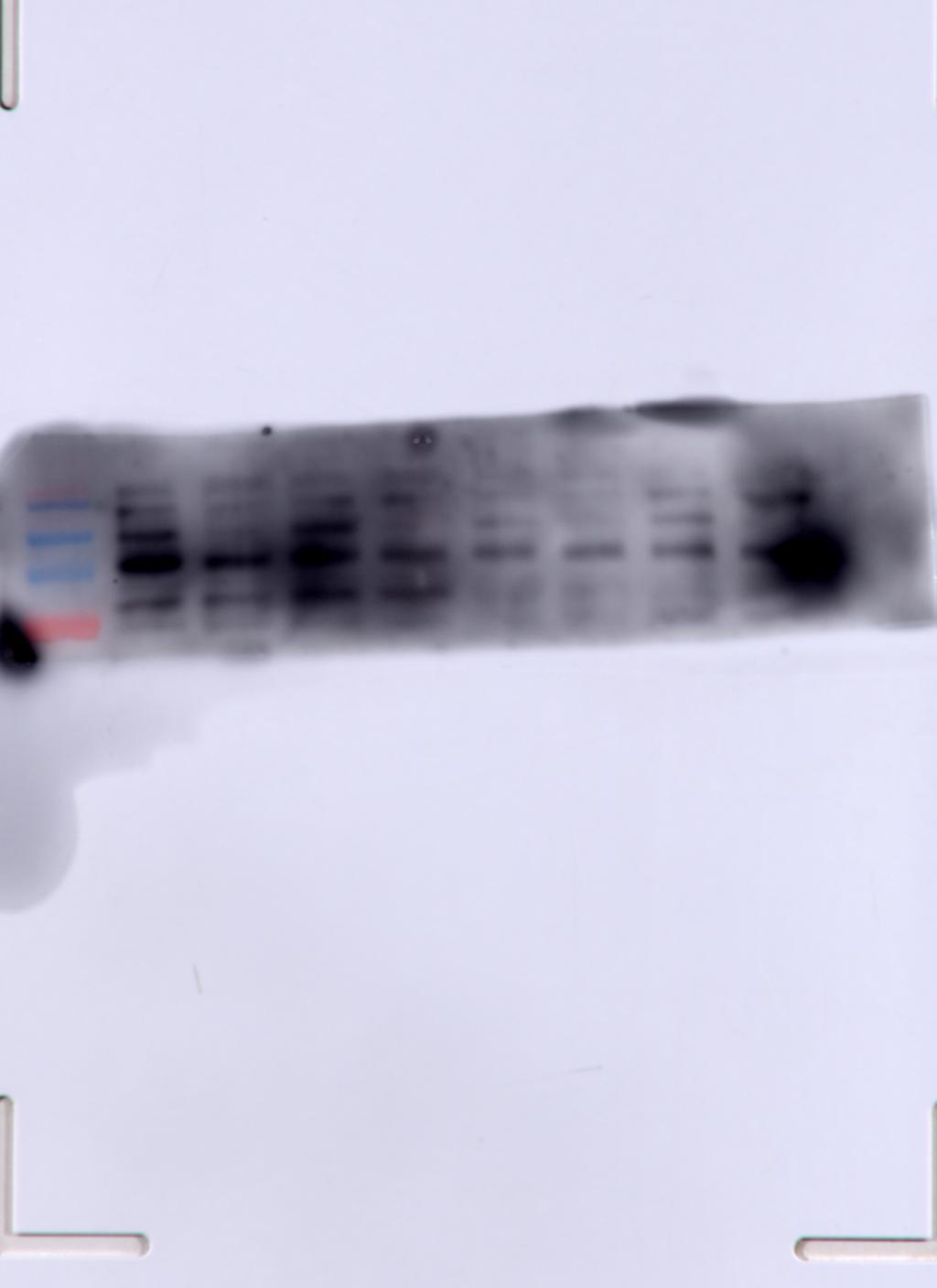

Supplement: Figure 7—source data 1. [file elife-87510-fig7-data1.zip › figure7-soure-data/Figure7B-zeb2.jpg]

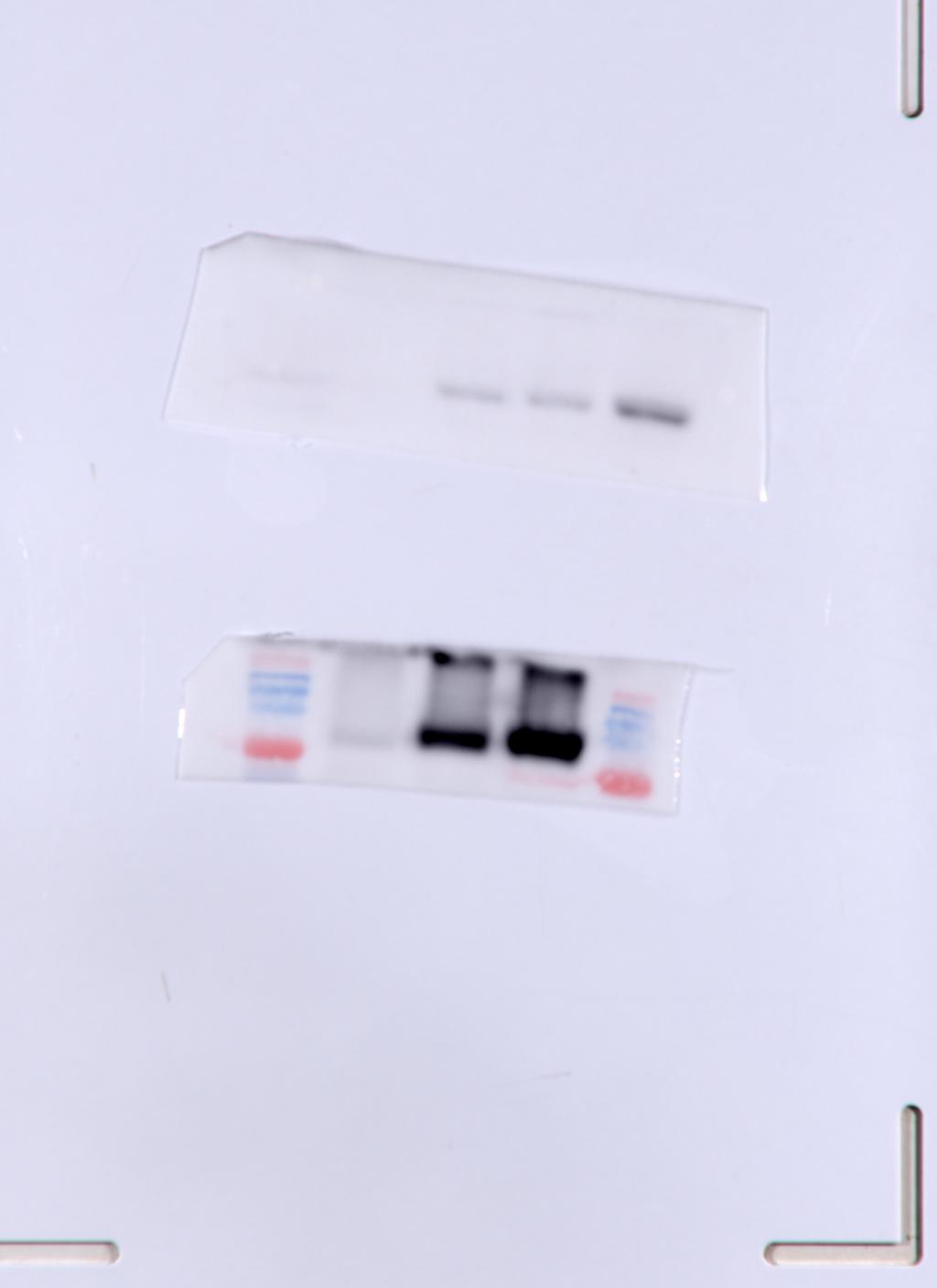

Supplement: Figure 7—source data 1. [file elife-87510-fig7-data1.zip › figure7-soure-data/Figure7C-ACSL4.jpg]

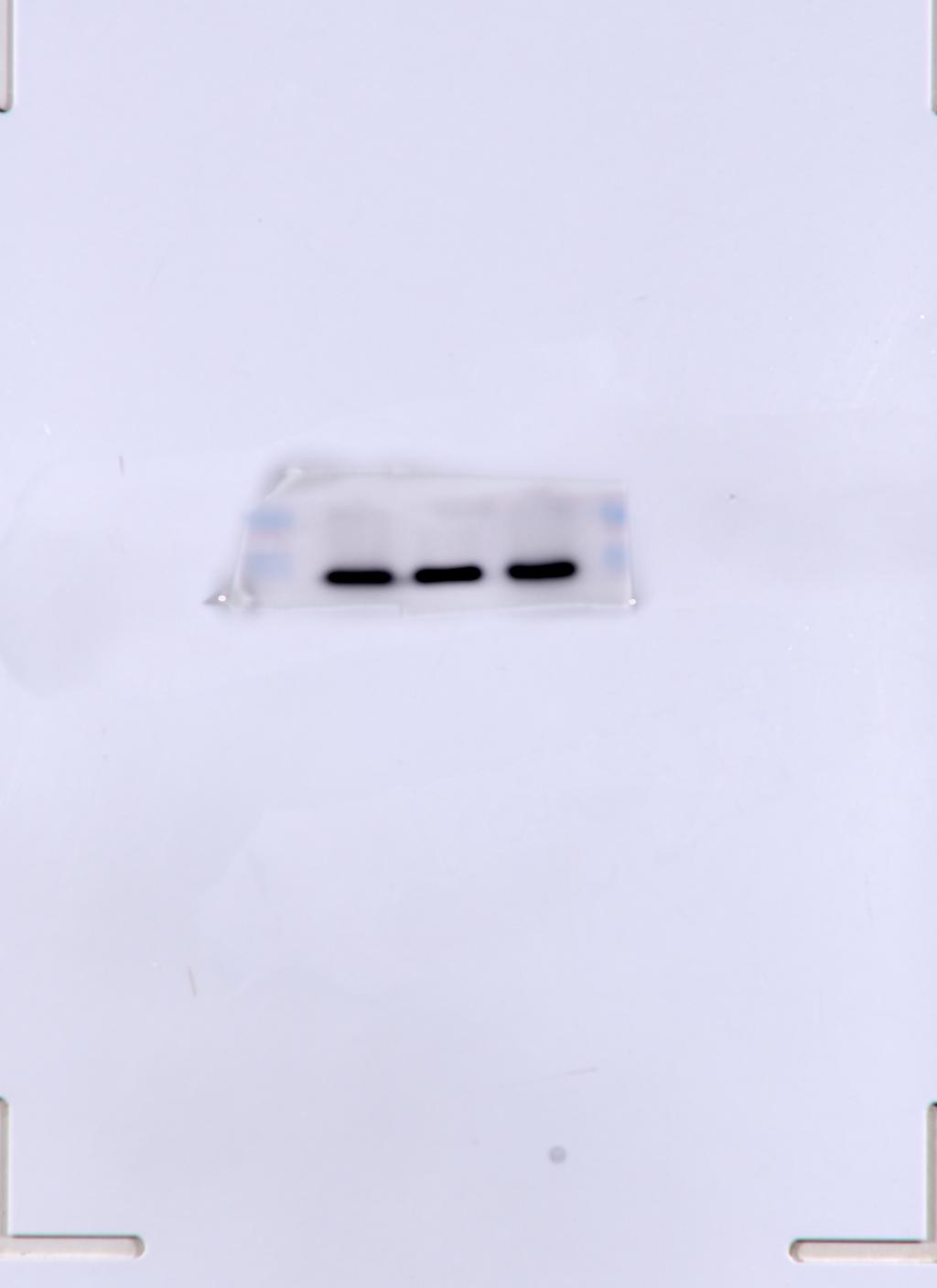

Supplement: Figure 7—source data 1. [file elife-87510-fig7-data1.zip › figure7-soure-data/Figure7C-gapdh-input .jpg]

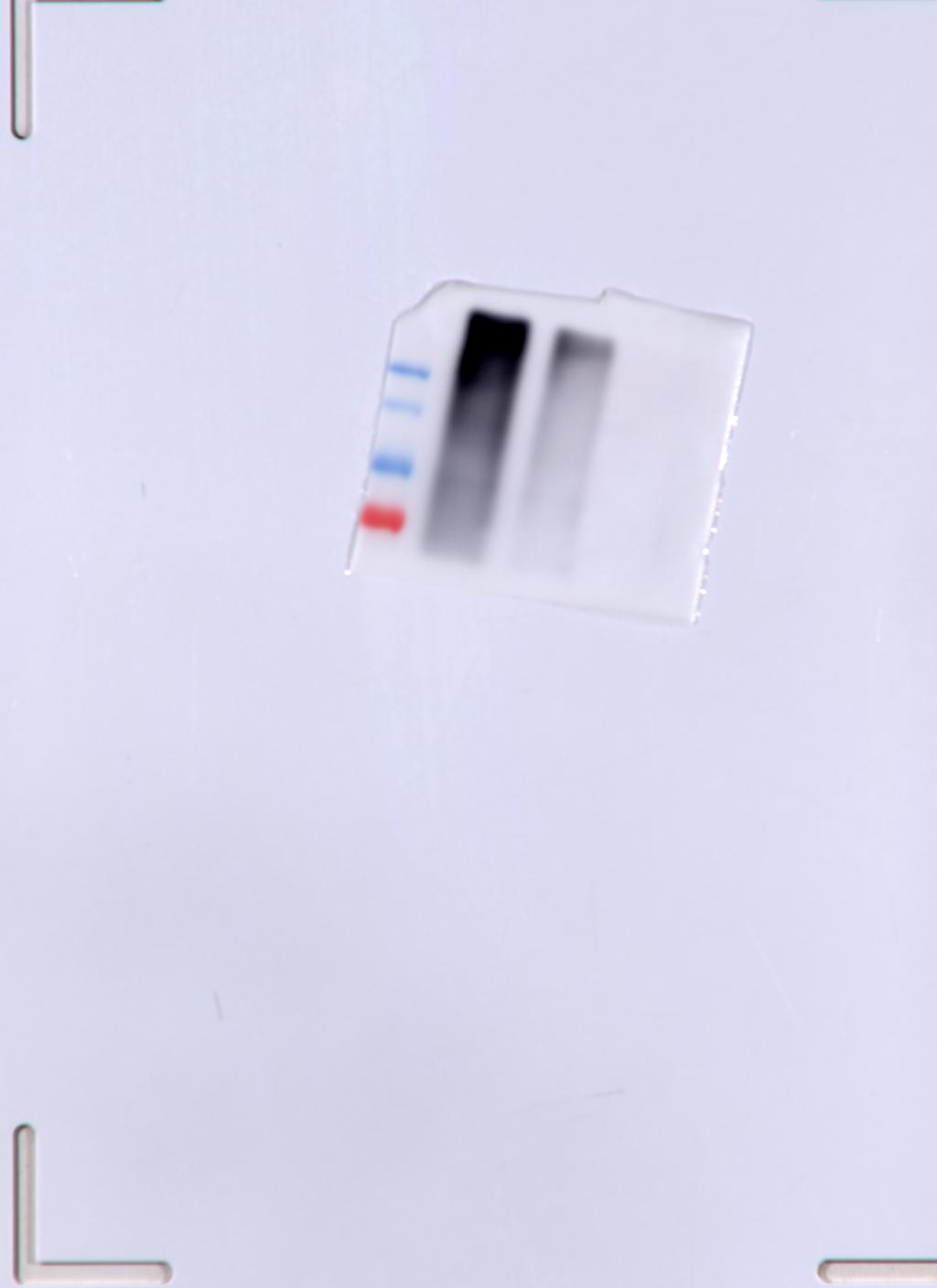

Supplement: Figure 7—source data 1. [file elife-87510-fig7-data1.zip › figure7-soure-data/Figure7C-ZEB2(IB-HA)-ip MYC.jpg]

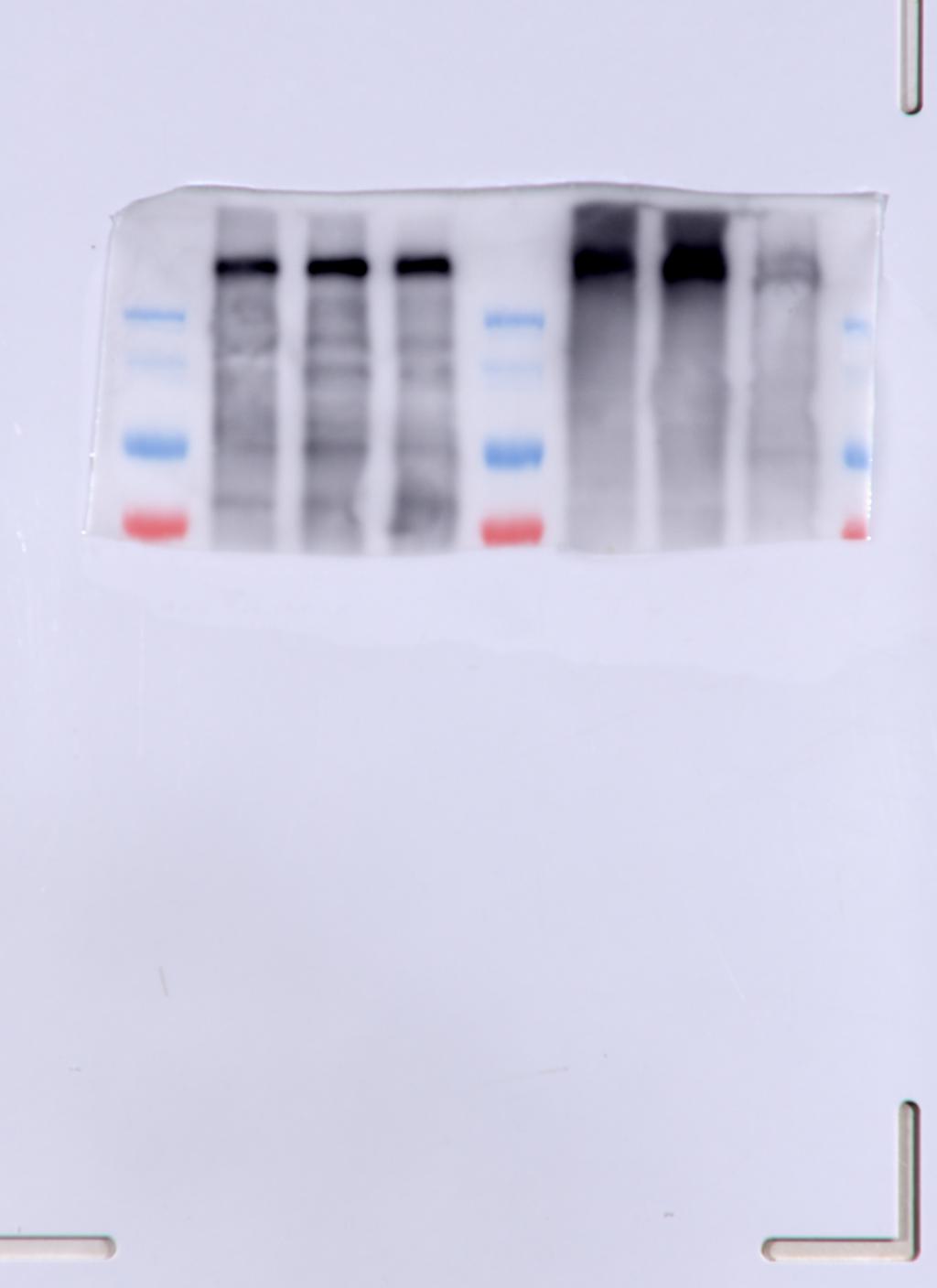

Supplement: Figure 7—source data 1. [file elife-87510-fig7-data1.zip › figure7-soure-data/Figure7C-ZEB2-myc .jpg]

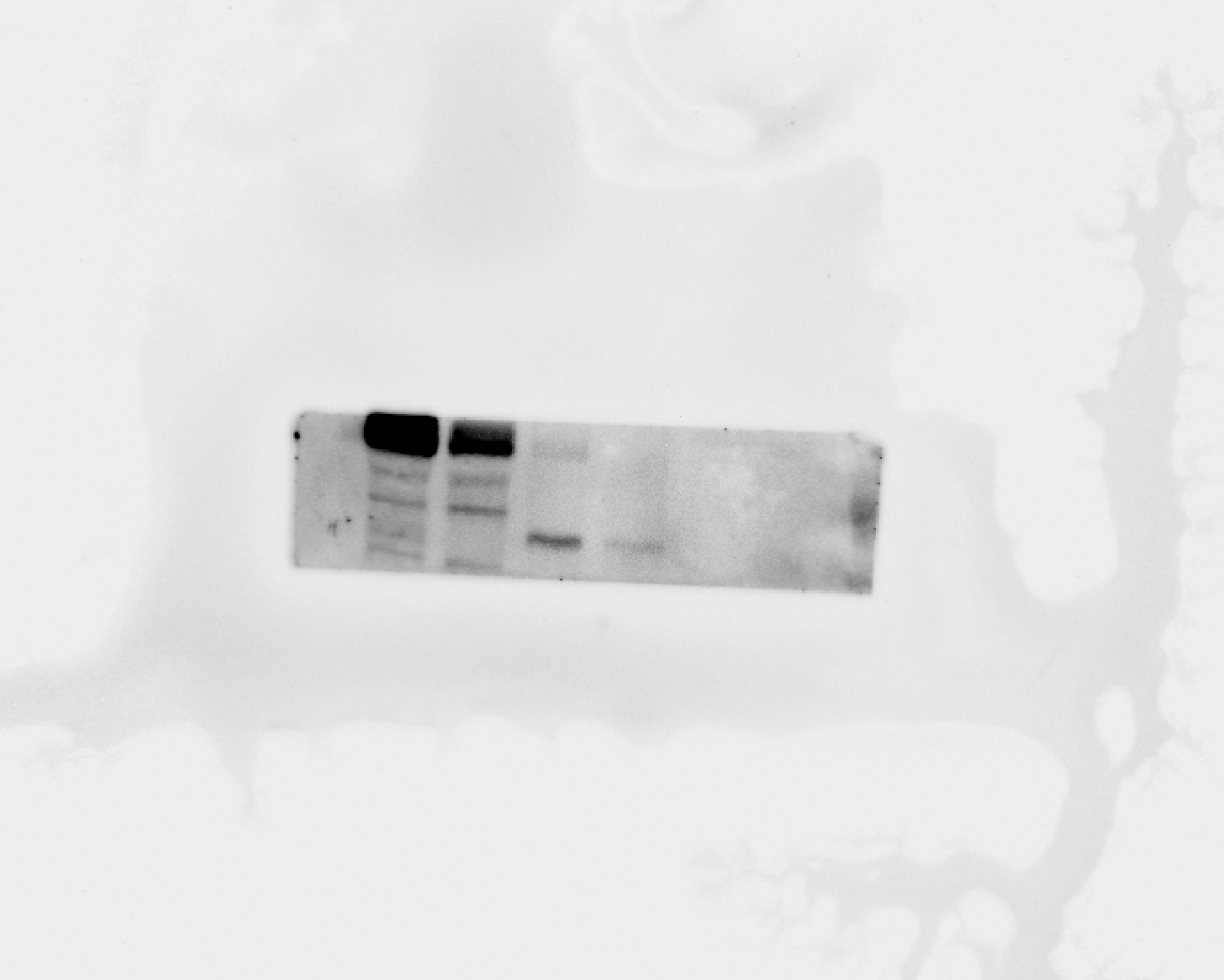

Supplement: Figure 7—source data 1. [file elife-87510-fig7-data1.zip › figure7-soure-data/Figure7D-ACSL4.jpg]

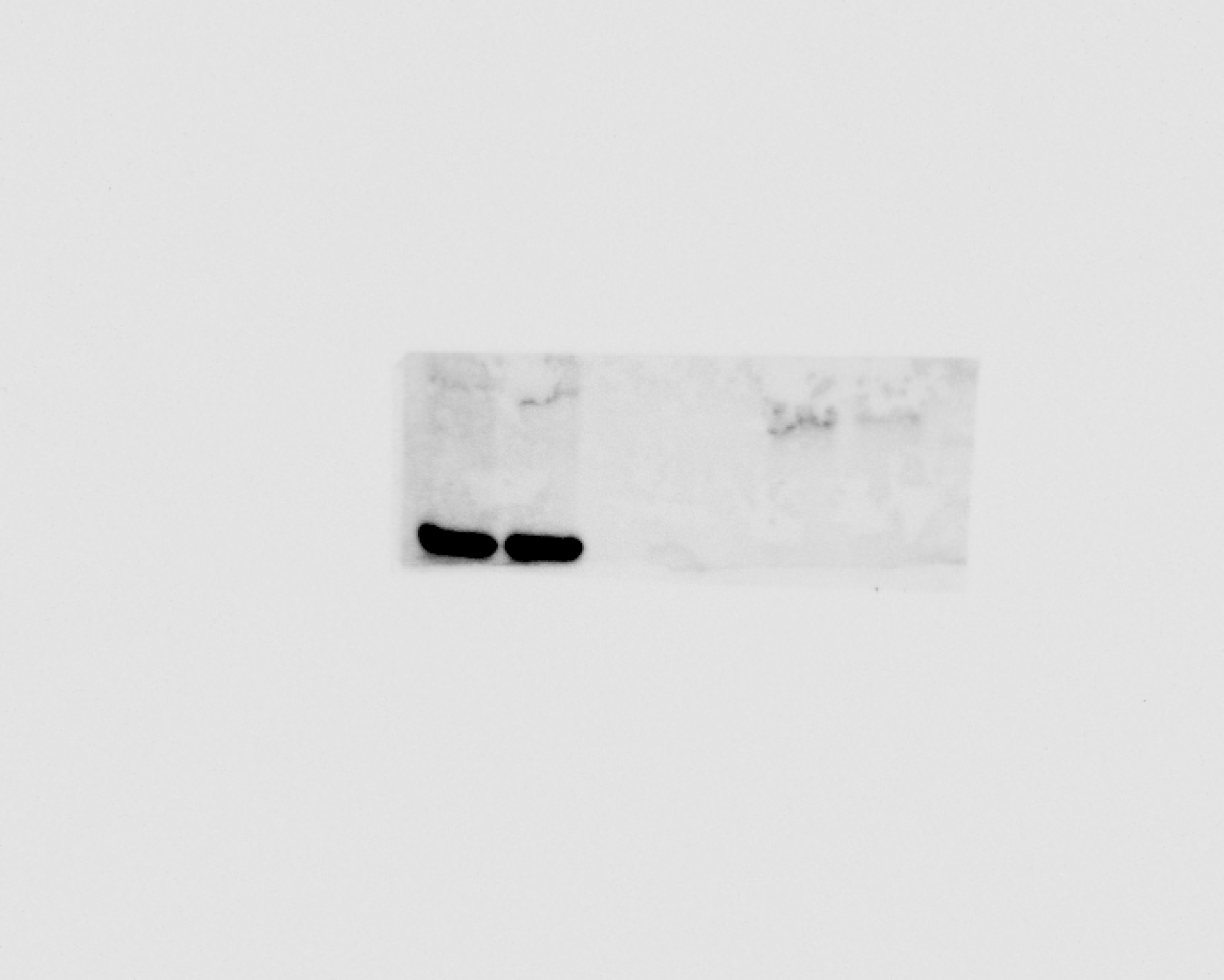

Supplement: Figure 7—source data 1. [file elife-87510-fig7-data1.zip › figure7-soure-data/Figure7D-GAPDH.jpg]

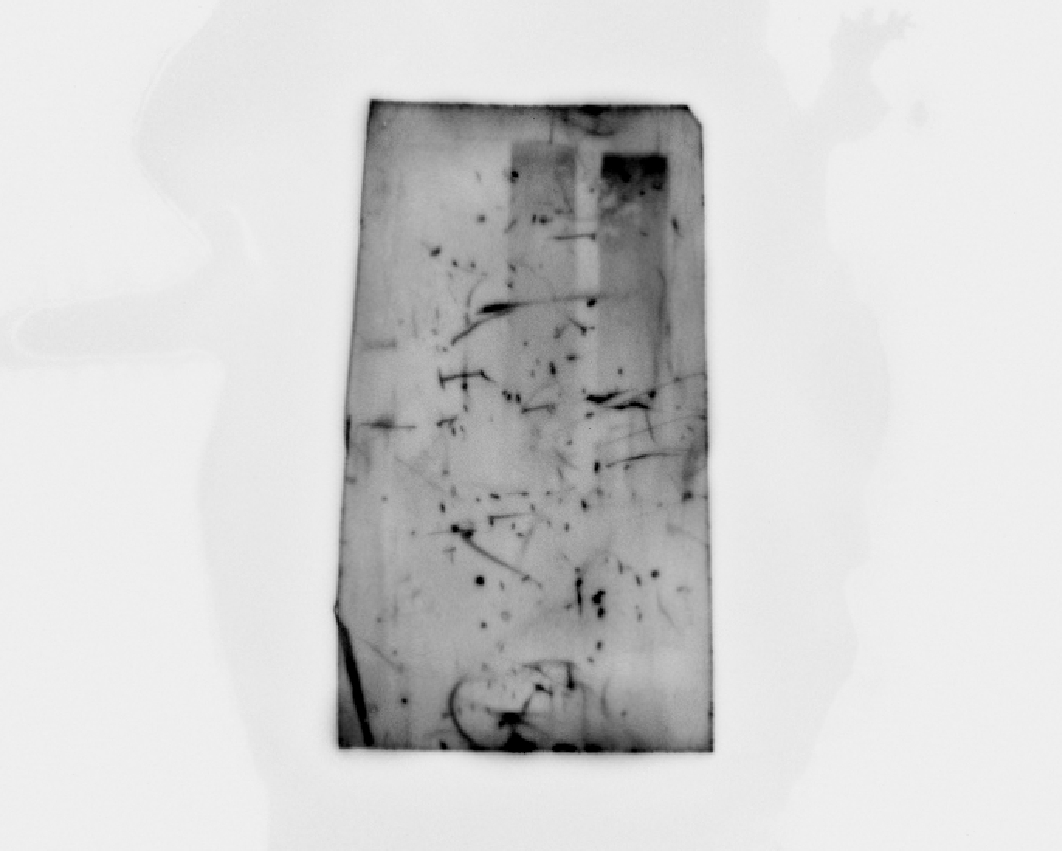

Supplement: Figure 7—source data 1. [file elife-87510-fig7-data1.zip › figure7-soure-data/Figure7D-Ubi.jpg]

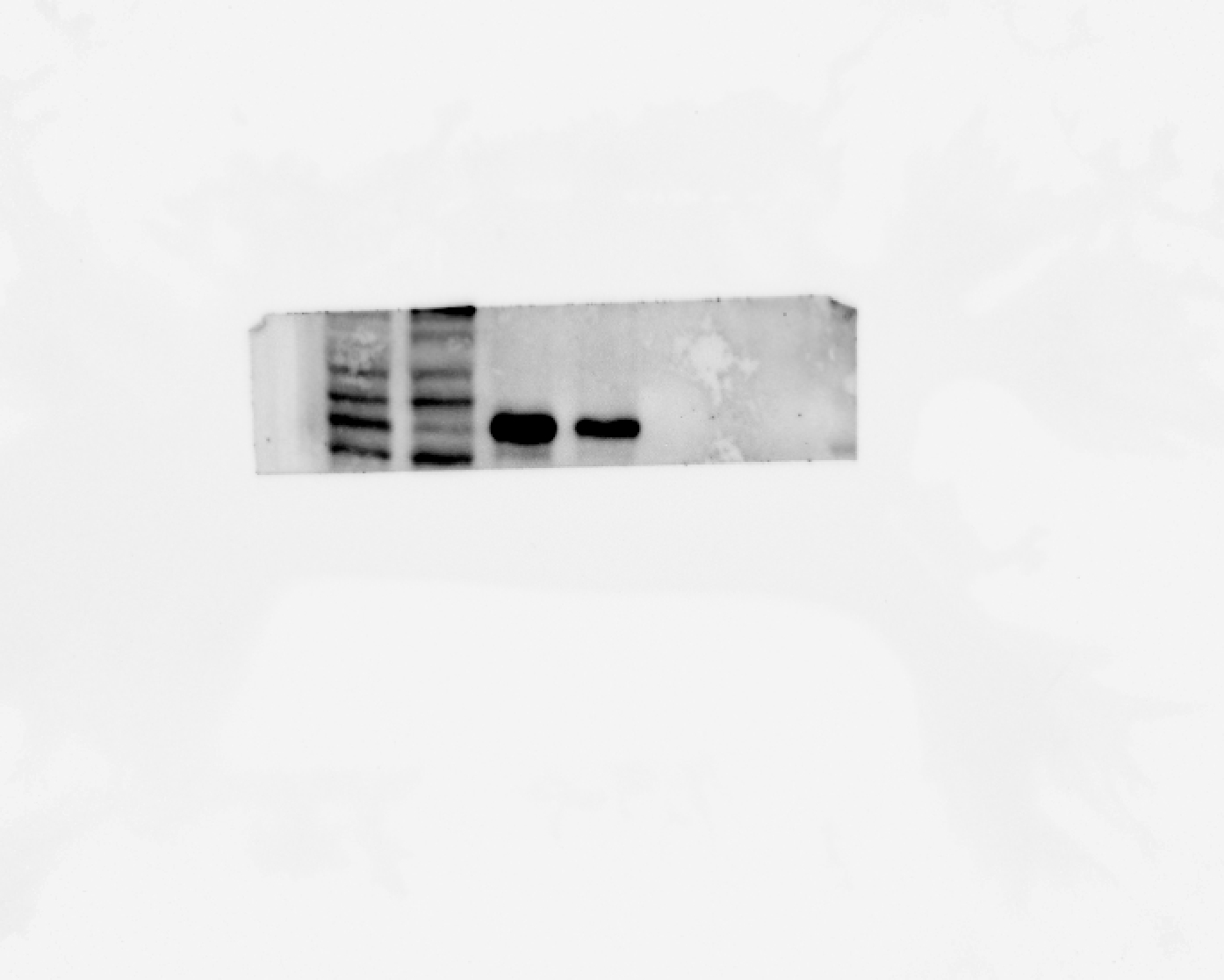

Supplement: Figure 7—source data 1. [file elife-87510-fig7-data1.zip › figure7-soure-data/Figure7D-ZEB2.jpg]

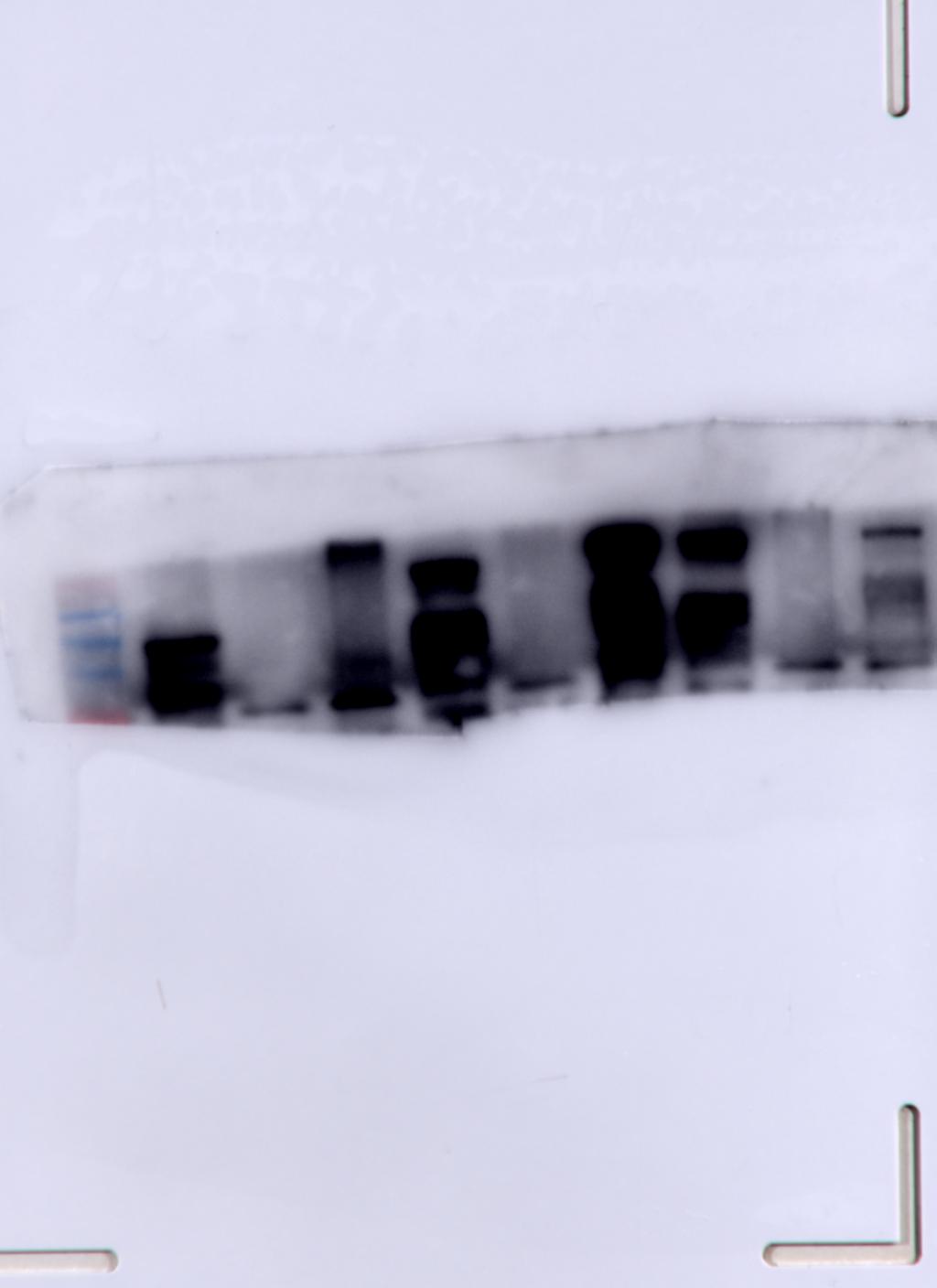

Supplement: Figure 7—source data 1. [file elife-87510-fig7-data1.zip › figure7-soure-data/Figure7E-ACSL4(ip-ACSL4).jpg]

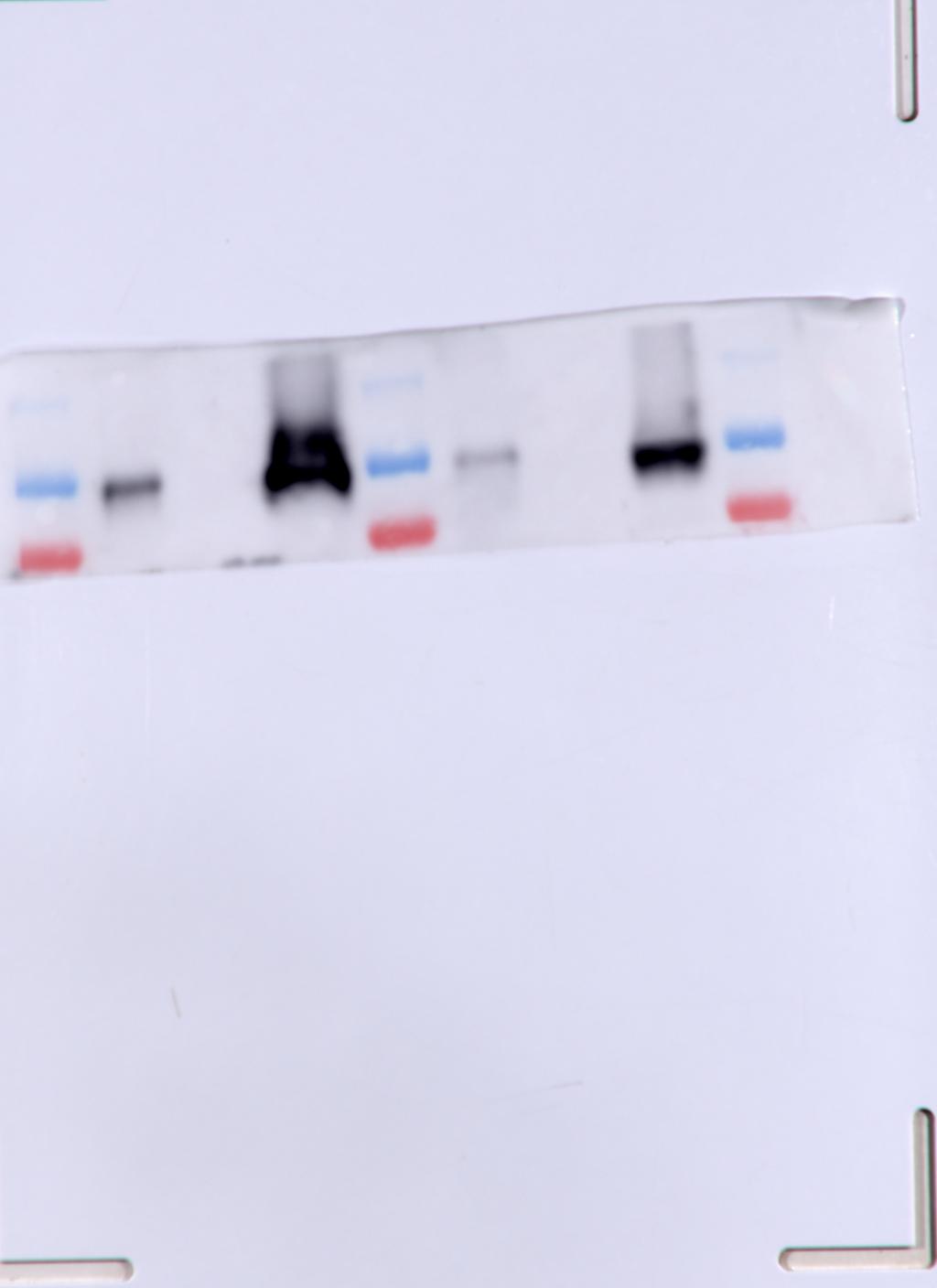

Supplement: Figure 7—source data 1. [file elife-87510-fig7-data1.zip › figure7-soure-data/Figure7E-ACSL4(ip-HA).jpg]

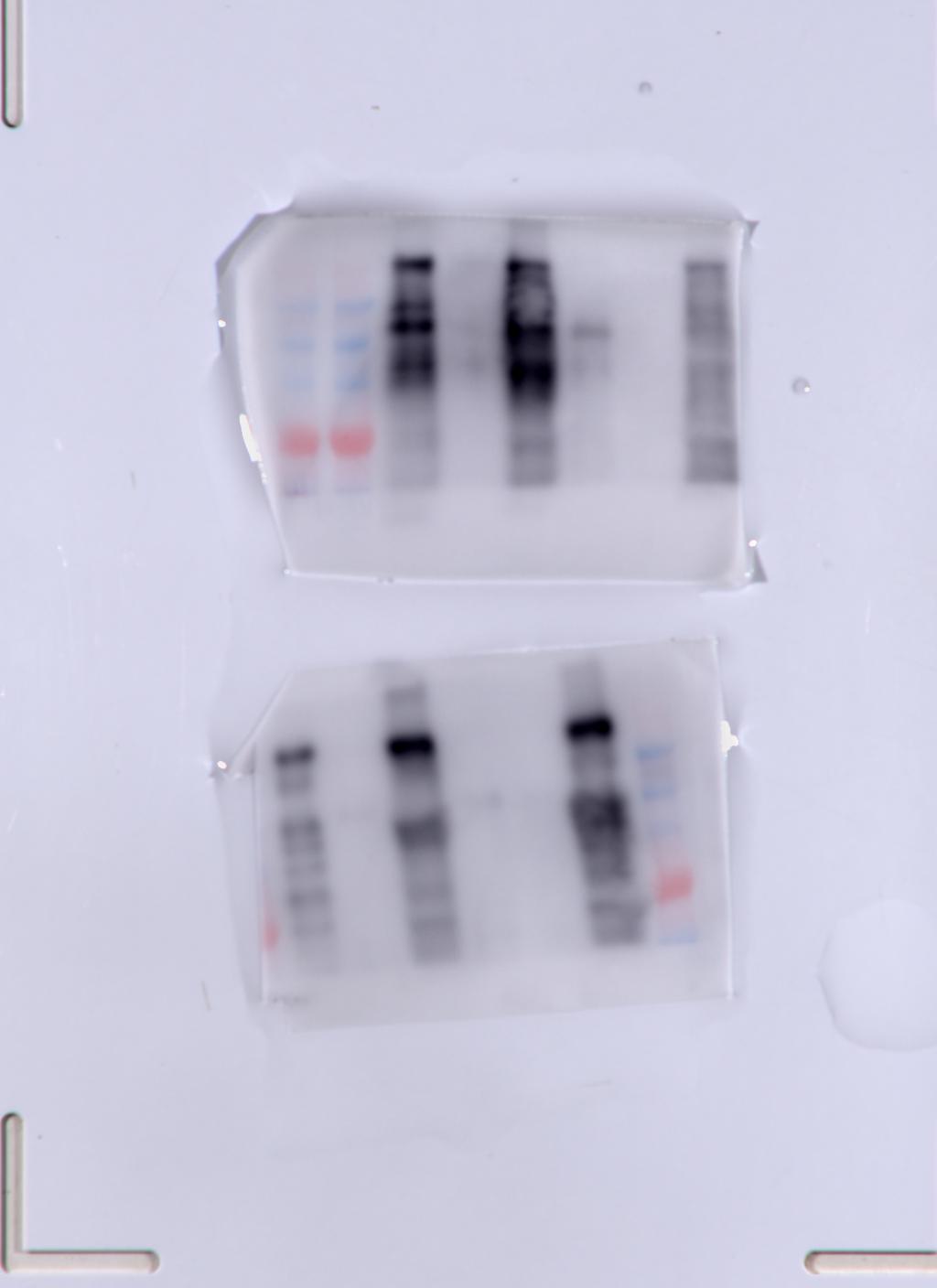

Supplement: Figure 7—source data 1. [file elife-87510-fig7-data1.zip › figure7-soure-data/Figure7E-ZEB2-HA(ip-ACSL4).jpg]

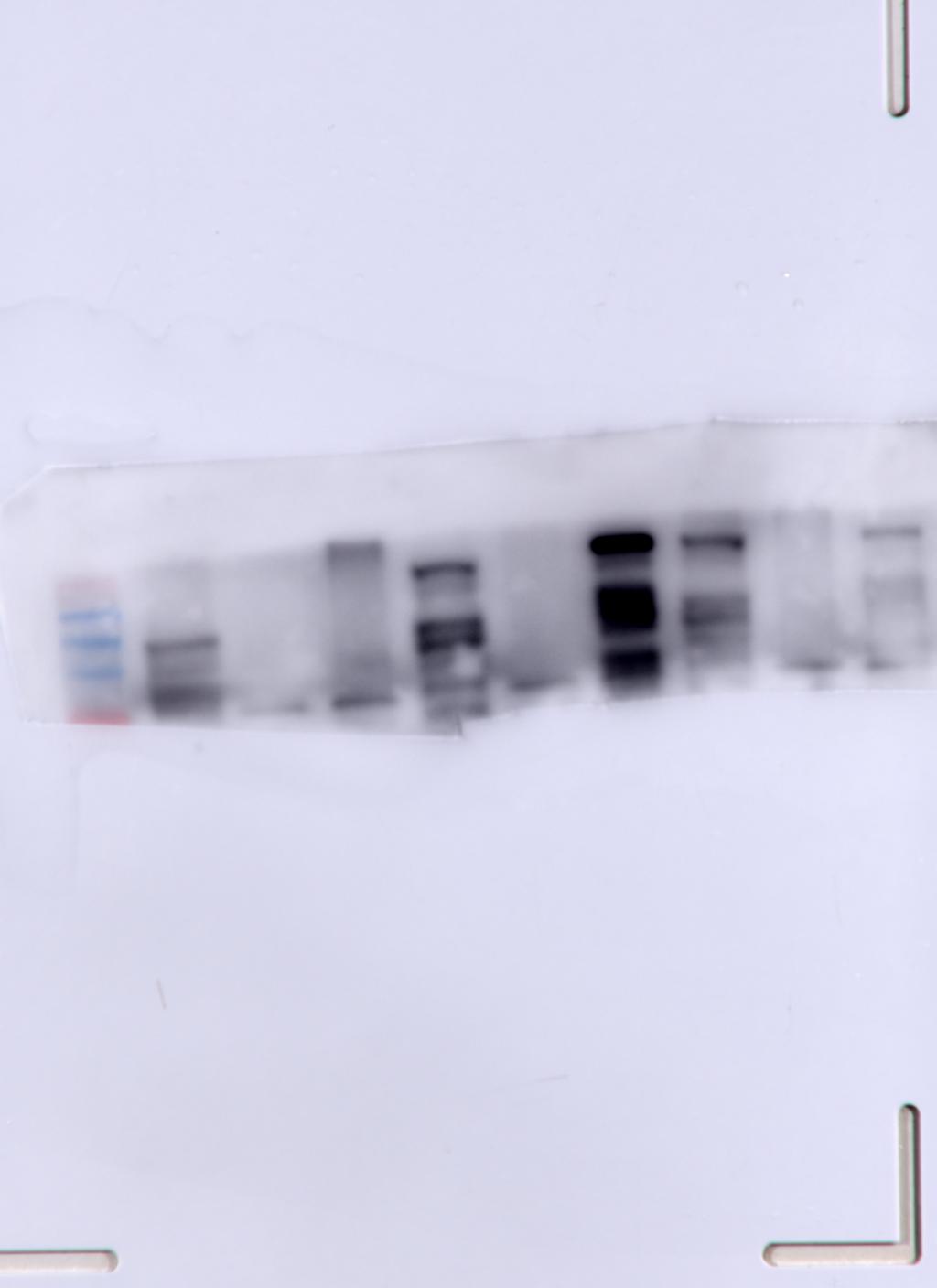

Supplement: Figure 7—source data 1. [file elife-87510-fig7-data1.zip › figure7-soure-data/Figure7E-ZEB2-HA(ip-HA).jpg]

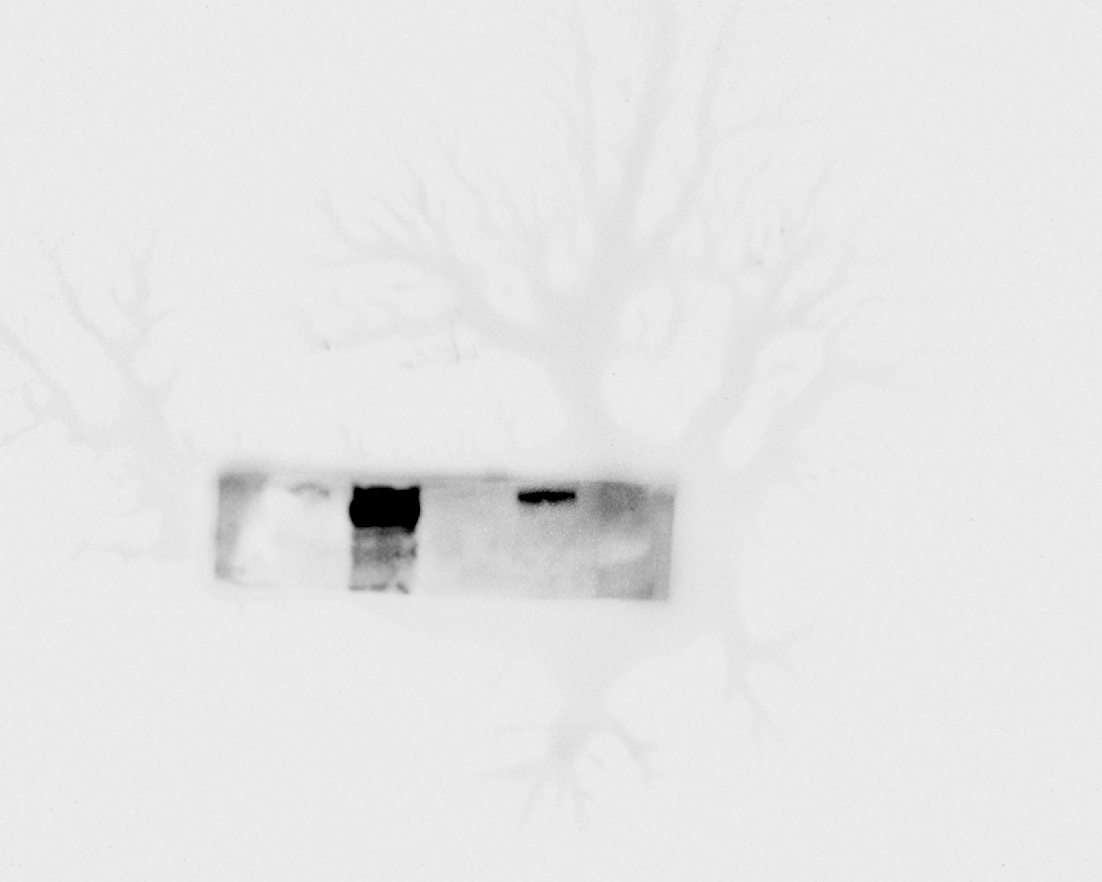

Supplement: Figure 7—source data 1. [file elife-87510-fig7-data1.zip › figure7-soure-data/Figure7f-ACSL4.tif]

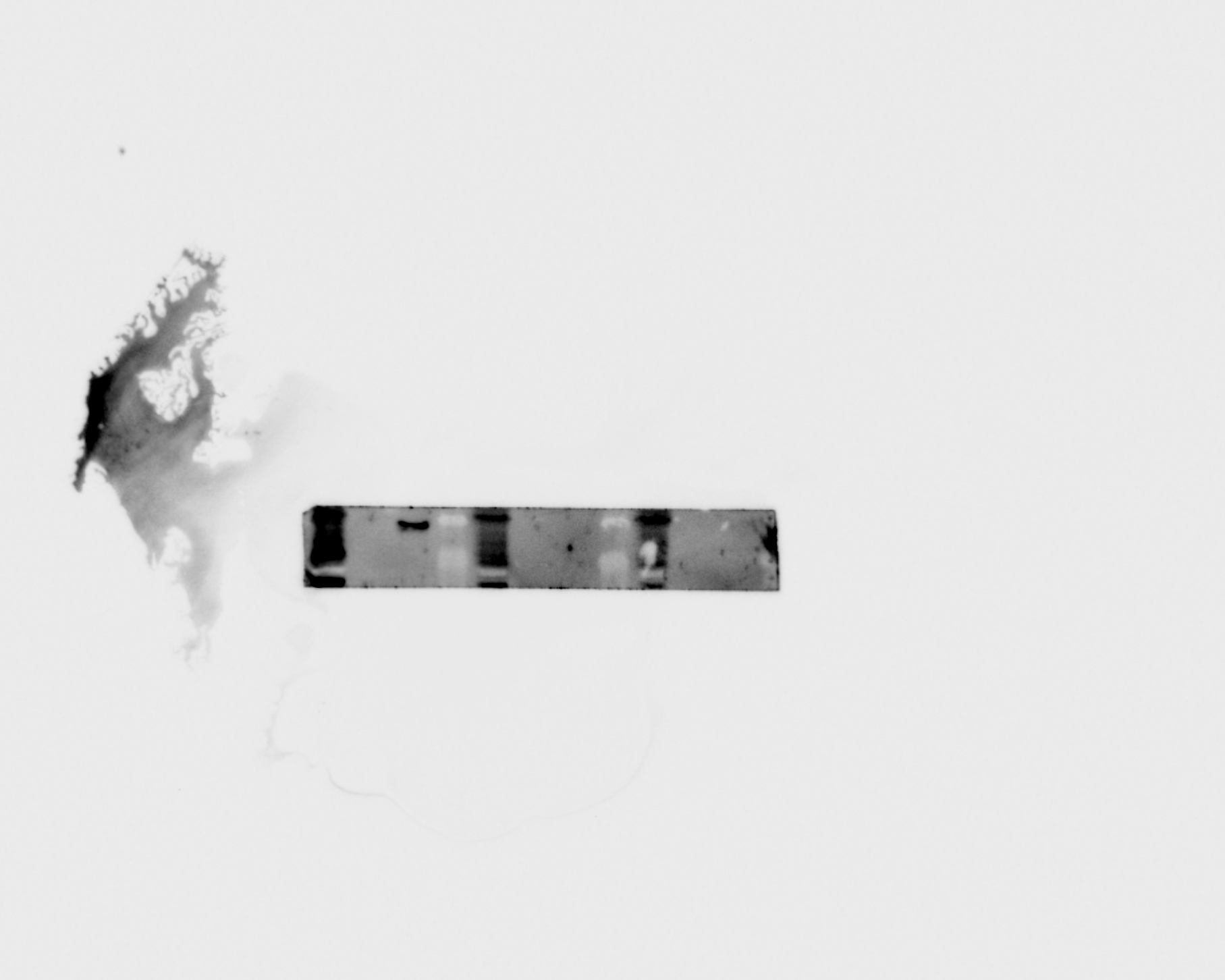

Supplement: Figure 7—source data 1. [file elife-87510-fig7-data1.zip › figure7-soure-data/Figure7f-ZEB2.jpg]

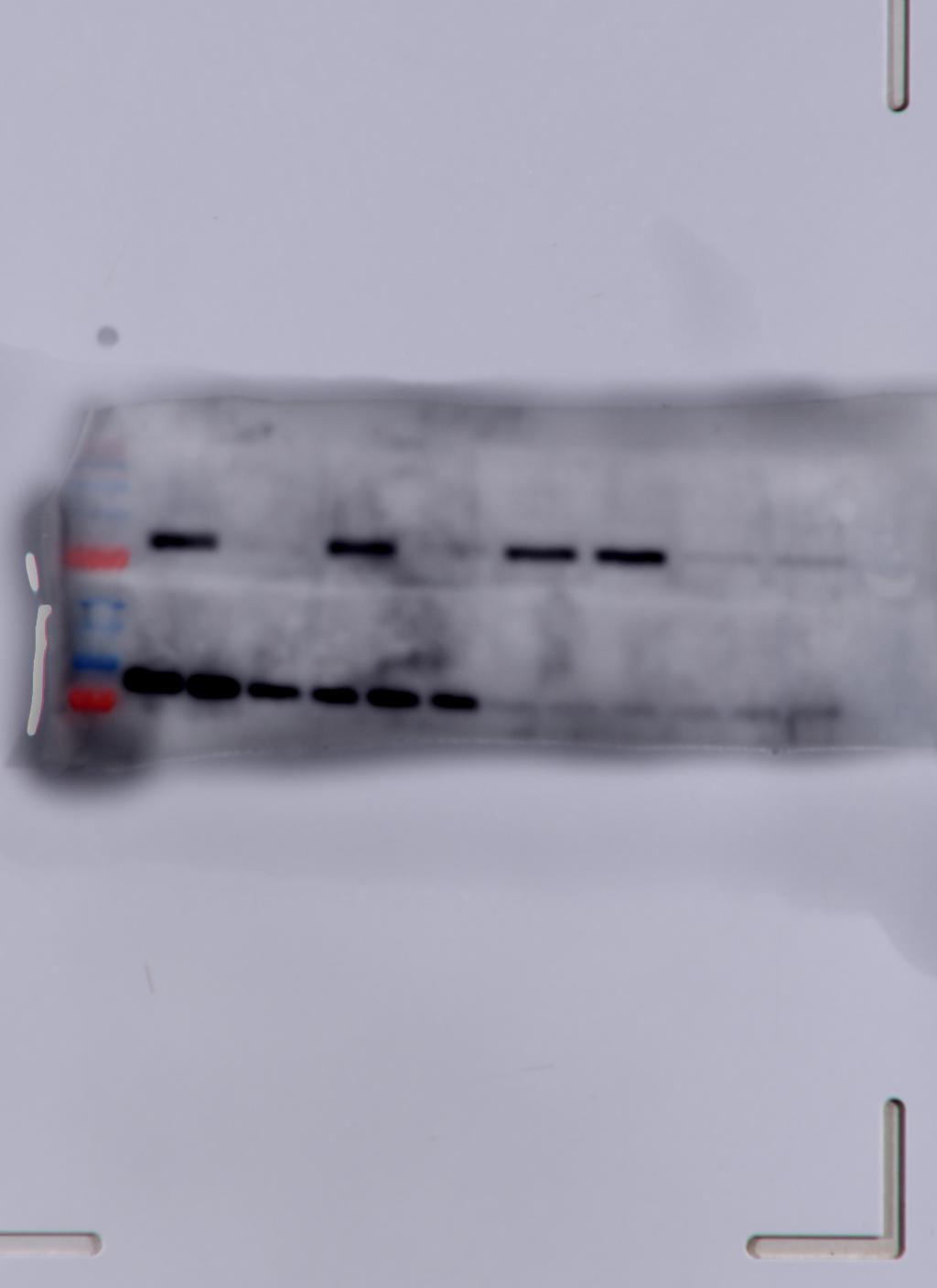

Supplement: Figure 7—source data 1. [file elife-87510-fig7-data1.zip › figure7-soure-data/Figure7G-ACSL4.jpg]

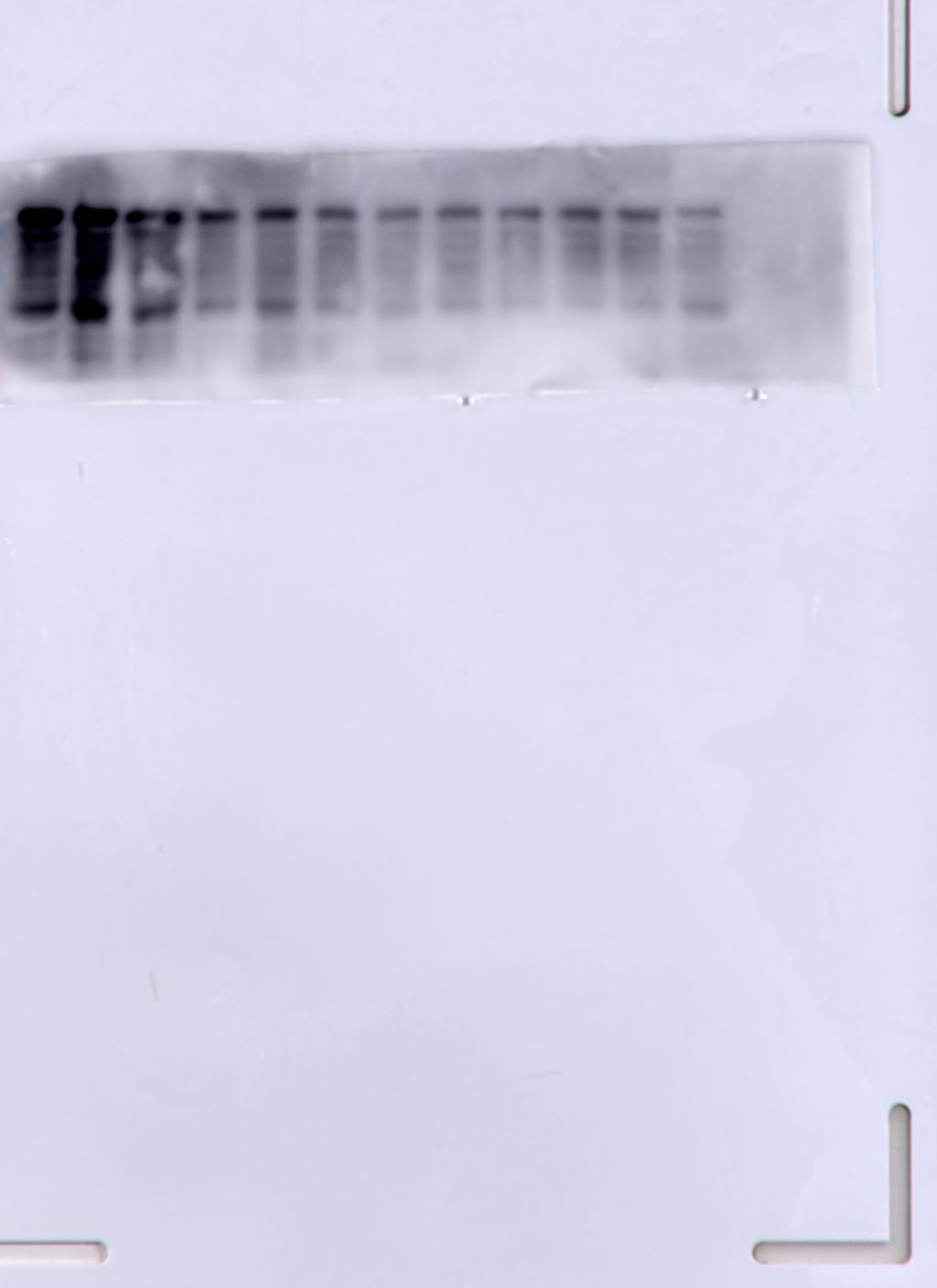

Supplement: Figure 7—source data 1. [file elife-87510-fig7-data1.zip › figure7-soure-data/Figure7G-ZEB2.jpg]

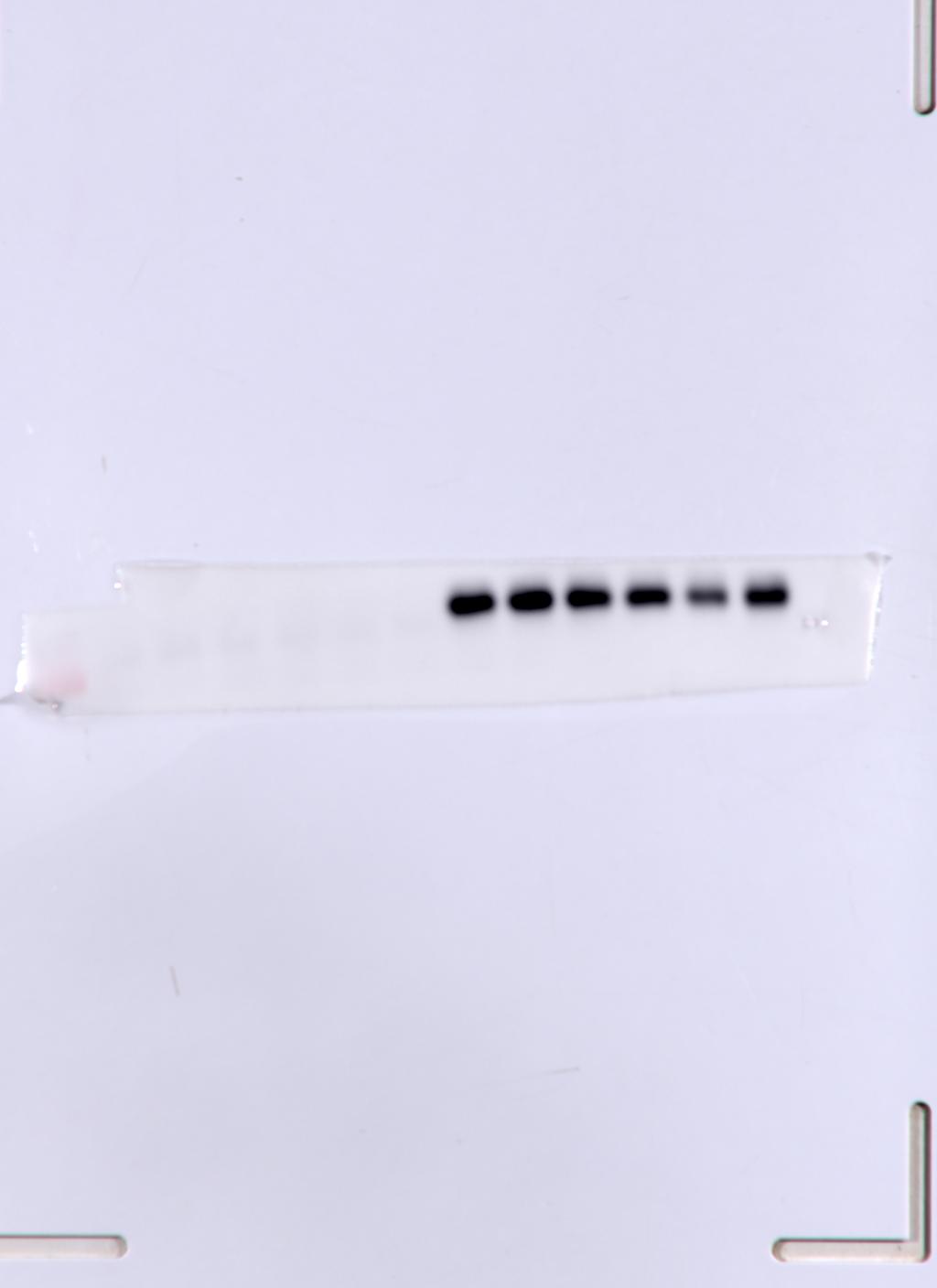

Supplement: Figure 7—source data 1. [file elife-87510-fig7-data1.zip › figure7-soure-data/Figure7I-ACSL4.jpg]

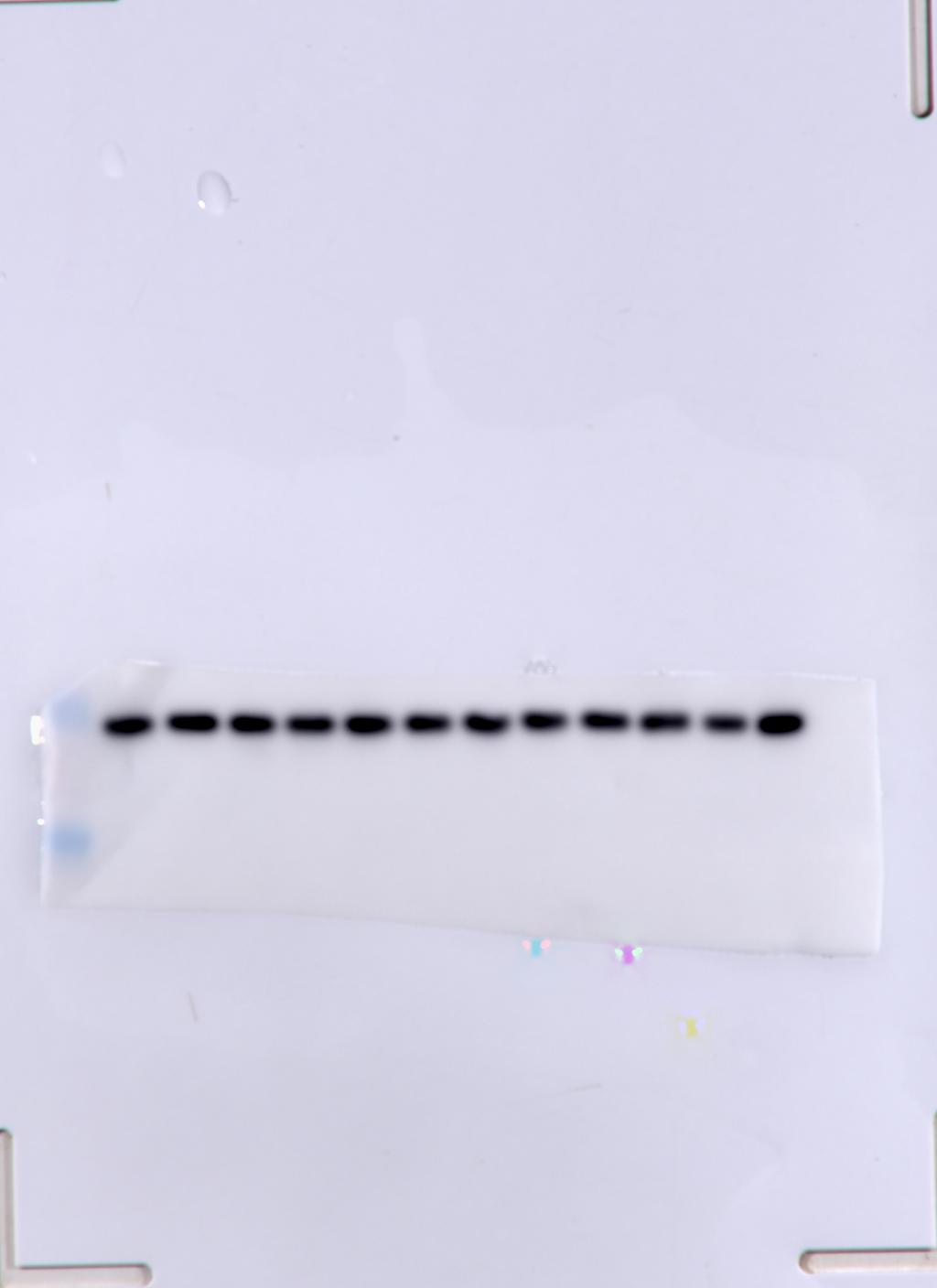

Supplement: Figure 7—source data 1. [file elife-87510-fig7-data1.zip › figure7-soure-data/Figure7I-gapdh.jpg]

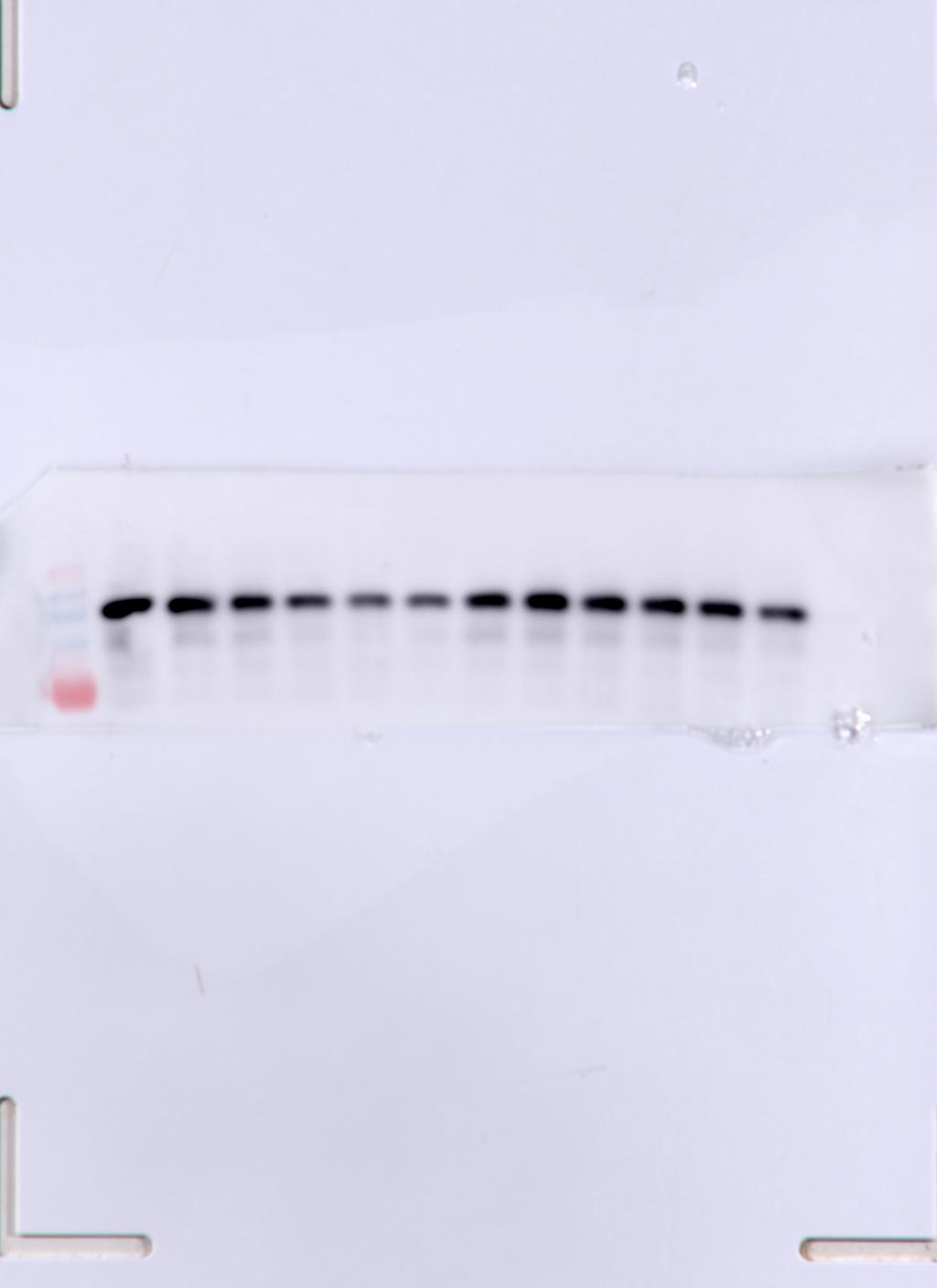

Supplement: Figure 7—source data 1. [file elife-87510-fig7-data1.zip › figure7-soure-data/Figure7I-Zeb2.jpg]

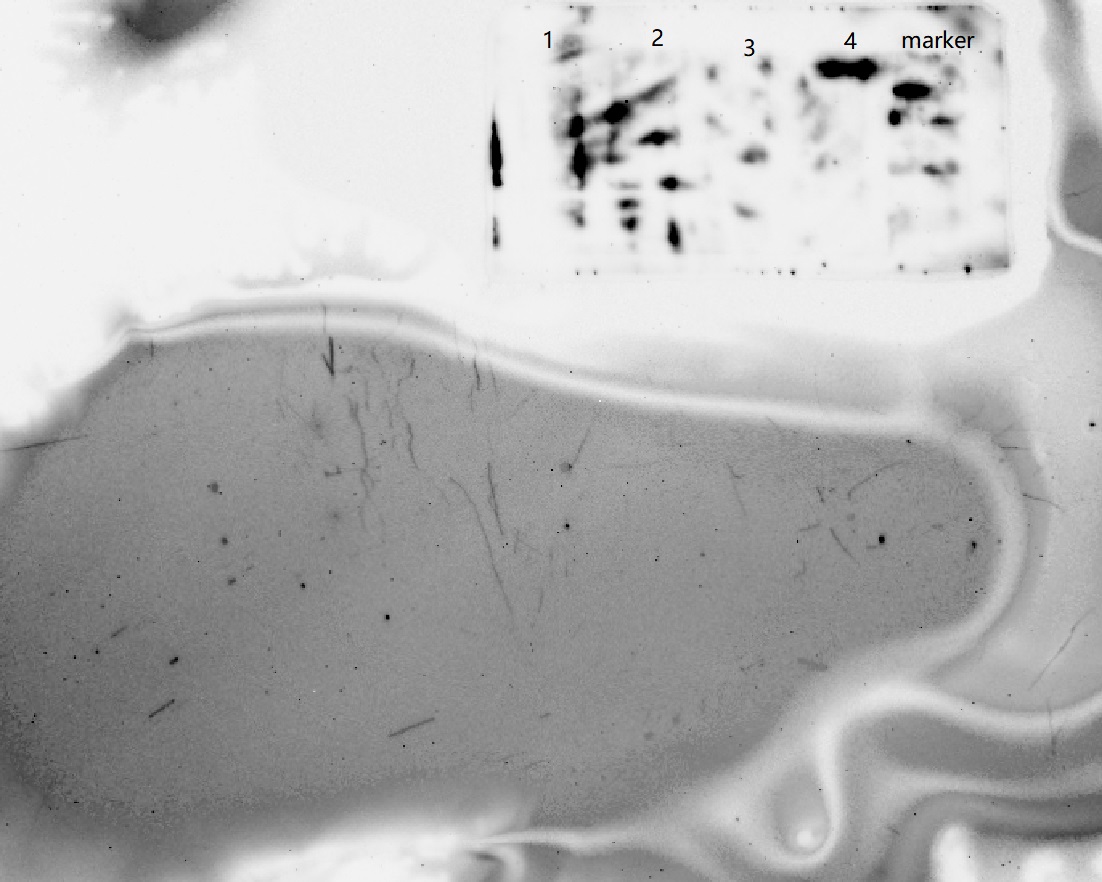

Supplement: Figure 7—source data 1. [file elife-87510-fig7-data1.zip › figure7-soure-data/ZEB2-MYC GST-pulldown .jpg]

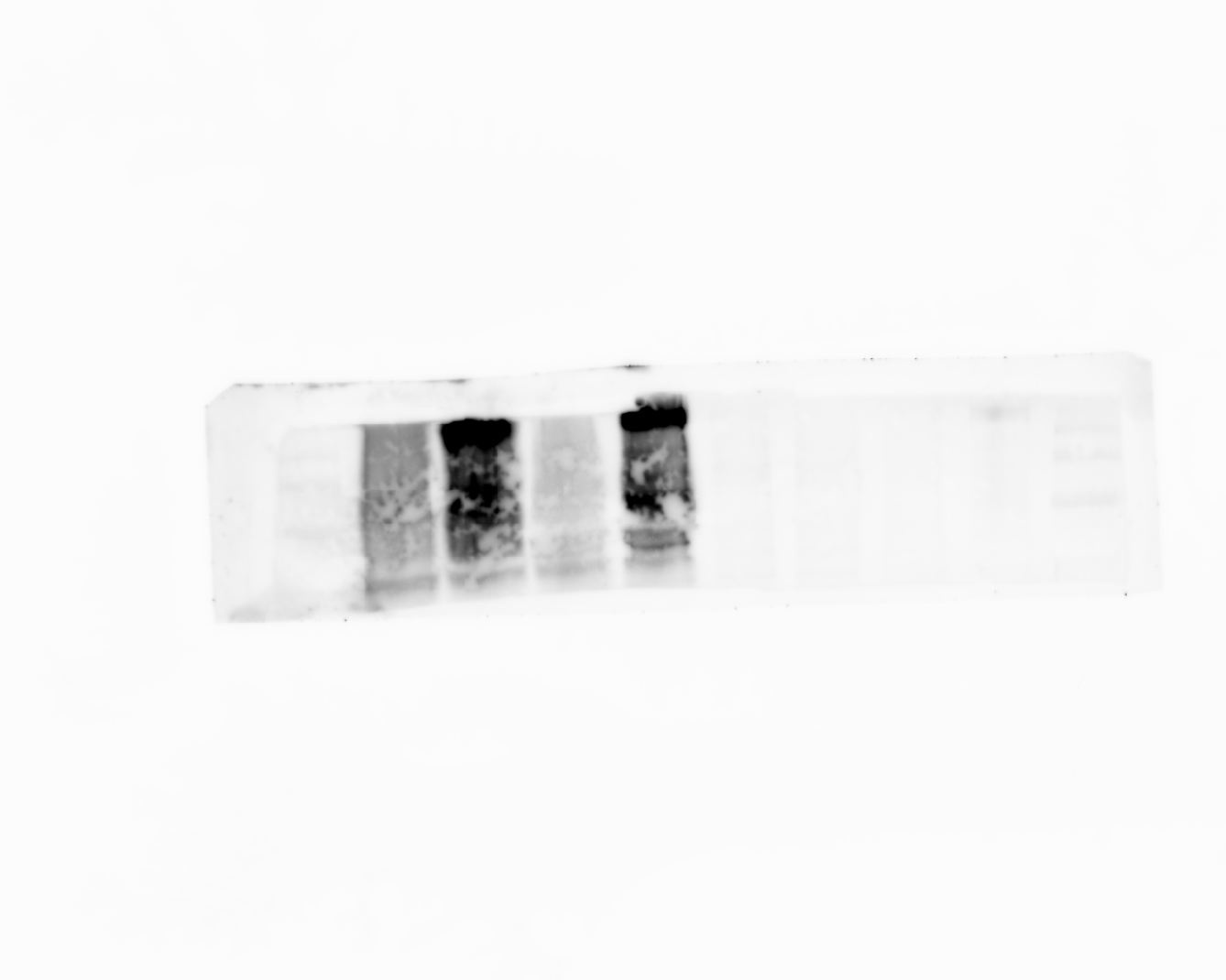

Supplement: Figure 7—source data 1. [file elife-87510-fig7-data1.zip › figure7-soure-data/ZEB2-MYC-input.tif]
