# Supplementary material for: A positive feedback loop between ZEB2 and ACSL4 regulates lipid metabolism to promote breast cancer metastasis
Source: eLife. 2023 Dec 11;12:RP87510. doi: 10.7554/eLife.87510 (PMC10712958; doi:10.7554/eLife.87510)
Supplement: Supplementary file 1. [file elife-87510-supp1.docx]

#### supplementary file 1a. The sequences of siRNA target

| Gene name |  | sequence |
| --- | --- | --- |
| ACSL4 | sense（5'-3'） | GAGCGATTTGAAATTCCAA |
| ACSL4 | antisense（5'-3'） | TTGGAATTTCAAATCGCTC |
| ZEB2 | sense（5'-3'） | GCACAUCAGCAGCAAGAAATT |
| ZEB2 | antisense（5'-3'） | UUUCUUGCUGCUGAUGUGCTT |
