# Supplementary material for: A positive feedback loop between ZEB2 and ACSL4 regulates lipid metabolism to promote breast cancer metastasis
Source: eLife. 2023 Dec 11;12:RP87510. doi: 10.7554/eLife.87510 (PMC10712958; doi:10.7554/eLife.87510)
Supplement: Supplementary file 2. [file elife-87510-supp2.docx]

**supplementary file 1b.** The sequences of gene-specific primers used for qRT-PCR

| Gene name |  | sequence |
| --- | --- | --- |
| ACSL4 | F-Primer | CATCCCTGGAGCAGATACTCT |
| ACSL4 | R-Primer | TCACTTAGGATTTCCCTGGTCC |
| ZEB2 | F-Primer | AATGCACAGAGTGTGGCAAGGC |
| ZEB2 | R-Primer | CTGCTGATGTGCGAACTGTAGG |
| GAPDH | F-Primer | GGAGCGAGATCCCTCCAAAAT |
| GAPDH | R-Primer | GGCTGTTGTCATACTTCTCATGG |
| CPT1A | F-Primer | GATCCTGGACAATACCTCGGAG |
| CPT1A | R-Primer | CTCCACAGCATCAAGAGACTGC |
| CPT1B | F-Primer | TGTATCGCCGTAAACTGGACCG |
| CPT1B | R-Primer | TGTCTGAGAGGTGCTGTAGCAC |
| CPT1C | F-Primer | TGCCATGTCGTTCCATTCTCCC |
| CPT1C | R-Primer | GCCGACTCATAAGTCAGGCAGA |
