# Supplementary material for: A positive feedback loop between ZEB2 and ACSL4 regulates lipid metabolism to promote breast cancer metastasis
Source: eLife. 2023 Dec 11;12:RP87510. doi: 10.7554/eLife.87510 (PMC10712958; doi:10.7554/eLife.87510)
Supplement: Supplementary file 3. [file elife-87510-supp3.docx]

**supplementary file 1c.** The control/-287bp/-965bp/-1036bp/-1116bp and -2000bp regions and motif1 sequences of primers used for the ACSL4 promoter vector constructs.

| Gene name | |  | sequence |
| --- | --- | --- | --- |
| seq1 | F-Primer | | GATAGGTACCGAGCTCTTACGCGTGCGA  GCGGGGGCG |
| seq2 | F-Primer | | GATAGGTACCGAGCTCTTACGCGTCAGG  TGAGGGCGTGGG |
| seq3 | F-Primer | | GATAGGTACCGAGCTCTTACGCGTTCAG  GTGGTAAGGCATTTTATATATACATATA  TATATACACACACACAA |
| seq4 | F-Primer | | GATAGGTACCGAGCTCTTACGCGTTCCA  GGTACCTACATTTCAACAAGCAC |
| seq5 | F-Primer | | GATAGGTACCGAGCTCTTACGCGTTTTA  GTGTCACCTGGGCTGCTTATTAAAAATTCA |
|  | R-Primer | | AGTACCGGAATGCCAAGCTTCCGGAATG  CCAAGCTTACTTAGA |
| mofit1 | F-Primer | | TACGCGTAAAAAGAGGGCGTGGGCCAAT  TCTGCGCCT |
|  | R-Primer | | CGCCCTCTTTTTACGCGTAAGAGCTCGGT  ACCTATCG |
