# Supplementary material for: A positive feedback loop between ZEB2 and ACSL4 regulates lipid metabolism to promote breast cancer metastasis
Source: eLife. 2023 Dec 11;12:RP87510. doi: 10.7554/eLife.87510 (PMC10712958; doi:10.7554/eLife.87510)
Supplement: Supplementary file 4. [file elife-87510-supp4.docx]

**supplementary file 1d** The sequences of gene-specific primers used for ChIP assay

| Gene name |  | sequence |
| --- | --- | --- |
| Set1-184bp/-295 | F-Primer | TCACCTGGGCTGCTTATT |
|  | R-Primer | GTGTGCATCACAATTATCTGGG |
| set2-784/-967bp | F-Primer | CTCCAGGTACCTACATTTCAA |
|  | R-Primer | TTGTGCTTGTGTGTGTGTATATATATATG |
| set3-912/-1046bp | F-Primer | CTCAGGTGGTAAGGCATTT |
|  | R-Primer | CCCAAAAAAATAAATCTCAAGAATTCTTCCA |
| set4—912/-1117bp | F-Primer | GCAAGCCGCAGGTGAGGGC |
|  | R-Primer | GATCCGCTTCTGTCAGTCTCGCTGC |
